# Chemical Constituents of *Vigna luteola* and Their Anti-inflammatory Bioactivity

**DOI:** 10.3390/molecules24071371

**Published:** 2019-04-08

**Authors:** Sio-Hong Lam, Yue-Chiun Li, Ping-Chung Kuo, Tsong-Long Hwang, Mei-Lin Yang, Chien-Chiao Wang, Jason T. C. Tzen

**Affiliations:** 1 School of Pharmacy, College of Medicine, National Cheng Kung University, Tainan 701, Taiwan; shlam@mail.ncku.edu.tw (S.-H.L.); L3891104@nckualumni.org.tw (M.-L.Y.); 2 Graduate Institute of Biotechnology, National Chung-Hsing University, Taichung 402, Taiwan; ycli0126@gmail.com; 3 Graduate Institute of Natural Products, College of Medicine, Chang Gung University, Taoyuan 333, Taiwan; htl@mail.cgu.edu.tw (T.-L.H.); D0501502@cgu.edu.tw (C.-C.W.); 4 Research Center for Industry of Human Ecology, Research Center for Chinese Herbal Medicine, and Graduate Institute of Health Industry Technology, Chang Gung University of Science and Technology, Taoyuan 333, Taiwan; 5 Department of Anesthesiology, Chang Gung Memorial Hospital, Taoyuan 333, Taiwan

**Keywords:** fabaceae, sesquiterpenoid, superoxide anion generation, elastase release

## Abstract

Seventy-three compounds were identified from the methanol extract of *V. luteola*, and among these, three new (**1**–**3**) were characterized by spectroscopic and mass spectrometric analyses. The isolated constituents were assessed for anti-inflammatory potential evaluation, and several purified principles exhibited significant superoxide anion and elastase inhibitory effects.

## 1. Introduction

Plenty of phytochemicals isolated from dietary and medicinal plants, such as epigallocatechin gallate [1], soy isoflavones [2], and curcumin [3,4], have been considered as promising sources of potential anticancer agents [5,6], and are one of the important sources for cancer treatment [7]. As of a thousand years ago, legumes had become essential for the protein supplements in the world. In addition, legumes are a considerable target for scientists to develop new foods, so that various seeds including the wild relatives and cultivated legumes attracted much attention. Many studies have reported that legumes possess various bioactivities [8,9,10]. Therefore, our team continued to explore edible wild beans as new natural health food supplements in the recent years.

Inflammation is the first response of the immune system to infection or irritation due to bacteria, virus, wound, or other various environmental factors resulting in injuring. Recently, the overexpression of neutrophils has been demonstrated to be related with various human diseases and causing serious threat to human health [11,12,13,14,15]. A response to diverse stimuli of the immune system is the activation of neutrophils to secrete a series of cytotoxins, such as superoxide anion and elastase [16]. Therefore, equilibrium of superoxide anion production and elastase release in infected tissues and organs is important. Furthermore, only a few available agents could directly modulate neutrophil proinflammatory responses in clinical practice. Chemoprevention is the idea of feeding natural sources to protect the human body from various diseases. Thus, utilization of anti-inflammatory health food supplements is important for the reduction of some diseases such as cancer.

*V. luteola* (Jacq.) Benth. is a trailing or twining herb belonging to the Vigna genus usually distributed in the tropical regions. In Taiwan, *V. luteola* is grown in the open seaside at elevation below 100 m and spread over the island [17]. The Vigna genus has been reported to show antioxidant [18,19], antifungal [20], antitumor [21,22], deworming [23,24], hypoglycemic [25,26], hepatoprotective [27,28], kidney protection [29,30], antibacterial [31,32], hypotensive [33,34], and hypolipidemic [35,36] bioactivities. In our previous research, the constituents and anti-inflammatory bioactivity of *V. vexillata* were investigated and the results exhibited potent inhibitory activity of superoxide generation and elastase release [37]. Preliminary bioassay data indicated that the methanol extract and fractions of *V. luteola* (at 10 μg/mL) also displayed significant superoxide and elastase inhibition by human neutrophils in response to *N*-formyl-l-methionyl-phenylalanine/cytochalasin B (fMLP/CB) (Table 1). Therefore, the present study aimed to characterize the chemical constituents and anti-inflammatory bioactivity of *V. luteola*. In total, seventy-three compounds were identified and, among these, one sesquiterpenoid (**1**), one alkaloid (**2**), and one α-pyranone (**3**) were characterized based on spectroscopic and spectrometric analyses. Moreover, some of the isolated compounds were evaluated for their inhibitory activity of superoxide anion generation and elastase release.

## 2. Results and Discussion

### 2.1. Purification and Characterization

The whole-plants of *V. luteola* were air-dried, powdered, and extracted with methanol under reflux. The methanol extract was concentrated under reduce pressure to give a deep brown syrup extract. This extract was partitioned with water and chloroform to provide two soluble layers, i.e., chloroform soluble and water soluble layers, respectively. With the assistance of a combination of conventional chromatographic techniques, one sesquiterpenoid viglutin (**1**), one alkaloid viglutoside (**2**), one α-pyranone viglutanone (**3**), along with two salts sodium phaseate (**4**) and sodium p-coumarate (**5**), were characterized and their structures were constructed from the nuclear magnetic resonance (NMR) spectral elucidation and mass (MS) spectrometric analysis. Moreover, sixty-eight known compounds, including eight sesquiterpenoids, loliolide (**6**), isololiolide (**7**), blumenol A (**8**), kiwiionol (**9**), (+)-(*S*)-dehydrovomifoliol (**10**), (6*S*,9*R*)-roseoside (**11**), (3*S*,5*R*,6*S*,7*E*,9*R*)-megastigman-7-en-3,6,9-triol (**12**), (3*S*,5*R*,6*R*,7*E*)-3,5,6-trihydroxymegastigman-7-en-9-one (**13**); two sesquiterpenoids, abscisic acid (**14**) and machilusoxide A (**15**); one diterpenoid, 3(17)-phytene-1,2-diol (**16**); two triterpenoids, lupeol (**17**) and simiarenol (**18**); seven steroids, mixture of β-sitosterol (**19**) and stigmasterol (**20**), mixture of β-sitosteryl-3-*O*-β-d-glucoside (**21**), and stigmasteryl-3-*O*-β-d-glucoside (**22**), mixture of stigmast-4-en-3-one (**23**) and stigmast-4,22-dien-3-one (**24**), ergosterol peroxide (**25**); one lignan, (+)-pinoresinol (**26**); eight alkaloids, uracil (**27**), uridine (**28**), 6-hydroxymethyl-3-pyridinol (**29**), thymine (**30**), adenine (**31**), nicotinamide (**32**), potassium nicotinate (**33**), 6-hydroxynicotinic acid (**34**); nineteen benzenoids, *p*-hydroxybenzoic acid (**35**), methylparaben (**36**), *trans*-methyl *p*-coumarate (**37**), (*E*)-methyl ferulate (**38**), *trans*-isoferulic acid (**39**), *trans*-ferulic acid (**40**), isovanillic acid (**41**), methyl vanillate (**42**), vanillic acid (**43**), 4-hydroxybenzaldehyde (**44**), syringic acid (**45**), gentisic acid (**46**), sodium salicylate (**47**), benzoic acid (**48**), methyl 2-*O*-β-d-glucopyranosylsalicylate (**49**), protocatechuic acid (**50**), phenylacetic acid (**51**), benzyl alcohol β-d-glucopyranosyl(1→6)-β-d-glucopyranoside (**52**), gentisic acid 5-*O*-β-d-xylopyranoside (**53**); nine flavonoids, kaempferol (**54**), liquiritigenin (**55**), kaempferol 3-*O*-[α-l-rhamnopyranosyl(1→6)]-β-d-galactopyranoside (**56**), chrysoeriol (**57**), astragalin (**58**), kaempferol-3-*O*-sophoroside (**59**), kaempferol 3-*O*-sophoroside-7-*O*-rhamnoside (**60**), robinin (**61**), isorhamnetin 3-*O*-[α-l-rhamnopyranosyl(1→6)]-β-d-galactopyranoside 7-*O*-α-l-rhamnopyranoside (**62**); eight isoflavonoids, daidzein (**63**), 8-*O*-methylretusin (**64**), 5,7,4′-trihydroxyisoflavone (**65**), 7,2′,4′-trihydroxyisoflavone (**66**), tectorigenin (**67**), afromosin (**68**), 3′-methoxydaidzein (**69**), 7,4′-dihydroxy-8-methoxyisoflavone (**70**); three others, 2-furanoic acid (**71**), maltol glucoside (**72**), sodium 5-hydroxymethylfuran-2-carboxylate (**73**), respectively, were verified by inspection of their reported spectroscopic data (references of known compounds were provided in Supplementary Materials).

### 2.2. Structural Elucidations of 1–5

**Figure 1:**
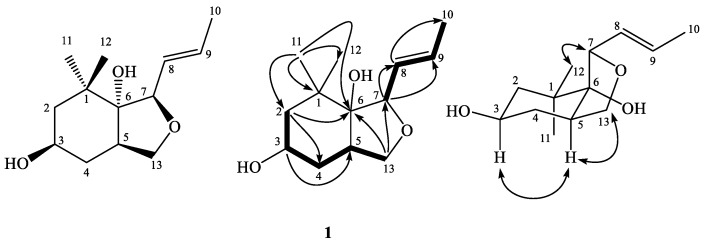
Key COSY (▬), HMBC (→), and NOESY (↔) correlations of **1**.

Compound **1** was isolated as optically active yellow powder, and the molecular formula was assigned as C_13_H_22_O_3_ with a sodium adduct ion peak at *m*/*z* 249.1460 in high-resolution electrospray ionization mass spectrometry (HR-ESI-MS) analysis. The infrared (IR) absorption bands at 3416 and 1647 cm^−^^1^ were in agreement with the presences of hydroxy and carbon–carbon double bond functionalities, respectively. In the ^1^H-NMR spectrum, there were proton signals for two methyl singlets at δ 0.96 (3H, s, CH_3_-11), 1.06 (3H, s, CH_3_-12), one methyl doublet of doublets 1.73 (3H, dd, *J* = 6.5, 1.5 Hz, CH_3_-10), two oxymethylene protons at δ 3.61 (1H, dd, *J* = 11.0, 7.0 Hz, H-13a) and 3.81 (1H, m, H-13b), two oxymethine at δ 3.80 (1H, dddd, *J* = 12.0, 7.5, 5.0, 3.5 Hz, H-3) and 4.18 (1H, d, *J* = 8.5 Hz, H-7), three methines at δ 1.36 (1H, ddd, *J* = 12.6, 5.0, 1.5 Hz, H-2b), 1.51 (1H, ddd, *J* = 12.0, 12.0, 11.5 Hz, H-4a), 2.44 (1H, m, H-5), and two trans olefinic protons at δ 5.67 (1H, ddd, *J* = 15.5, 8.5, 1.5 Hz, H-8) and 5.78 (1H, dq, *J* = 15.5, 6.5 Hz, H-9). The ^13^C-NMR and distortionless enhancement by polarization transfer (DEPT) spectra exhibited three methyl carbons at δ 18.2 (C-10), 24.0 (C-12) and 25.9 (C-11); three methylene carbons at δ 31.6 (C-4), 46.8 (C-2), and 69.7 (C-13); five methines at δ 43.7 (C-5), 68.5 (C-3), 84.4 (C-7), 129.6 (C-8), and 132.1 (C-9); and two quaternary carbons at δ 37.7 (C-1) and 81.2 (C-6). The correlation spectroscopy (COSY) indicated the linkage of H-2/H-3/H-4/H-5/H-13 and H-7/H-8/H-9/H-10 (Figure 1). The ^2^*J*- and ^3^*J*- correlation peaks from H-2 to C-4 and C-6; from H-3 to C-5; from H-7 to C-8 and C-9; from H-8 to C-10; from H-13 to C-6 and C-7; and from CH_3_-11 to C-1, C-2, C-6, and CH_3_-12, respectively, were observed in the 2-dimensions (2D) heteronuclear multiple bond correlation (HMBC) spectrum (Figure 1). In addition, the index of hydrogen deficiency (IHD = 3) of **1** suggested the presences of one double bond and two rings in **1**. According to this spectral evidence, the planar structure of **1** was constructed as shown (Figure 1). Furthermore, the large coupling constant of H-3 (12.0 Hz) revealed its axial orientation, and the correlations of H-3/H-5, H-5/H-13, and CH_3_-12/H-7 in the Nuclear Overhauser Effect spectroscopy (NOESY) established its relative stereochemistry configuration (Figure 1). All the proton and carbon signals assignments were achieved by the combination of 2D NMR experiments (see Supplementary Materials). Conclusively, the structure of **1** was assigned as shown based on the experimental results which mentioned above and named trivially as viglutin.

**Figure 2:**
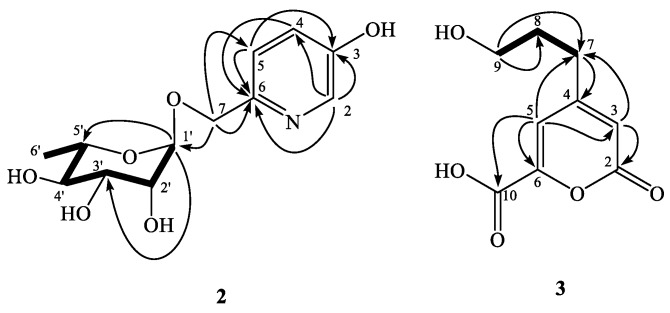
Key COSY (▬) and HMBC (→) correlations of **2** and **3**.

Compound **2** was assigned a molecular formula of C_12_H_17_NO_6_ from its HR-ESI-MS analytical data (*m/z* 294.0952, calc. for C_12_H_17_NNaO_6_, 294.0954). The UV absorption maxima at 274 and 221 nm were indication of the heteroaromatic chromophore [38], and it was further supported by the downfield ABX-coupled aromatic protons at δ 7.21 (1H, dd, *J* = 8.5, 2.5 Hz, H-4), 7.32 (1H, d, *J* = 8.5 Hz, H-5), and 8.03 (1H, d, *J* = 2.5 Hz, H-2). The existence of hydroxyl and carbon–carbon double bond functionalities could be determined from the IR absorption bands at 3430 and 1637 cm^−^^1^. One methylene group at δ 4.51 (1H, d, *J* = 12.0 Hz, H-7a) and 4.65 (1H, d, *J* = 12.0 Hz, H-7b), and one set of rhamnose protons at δ 1.25 (3H, d, *J* = 6.0 Hz, H-6’), 3.38 (1H, dd, *J* = 9.5, 9.5 Hz, H-4’), 3.60 (1H, dd, *J* = 9.5, 6.0 Hz, H-5’), 3.67 (1H, dd, *J* = 9.5, 3.5 Hz, H-3’), 3.85 (1H, dd, *J* = 3.5, 2.0 Hz, H-2’), and 4.78 (1H, d, *J* = 2.0 Hz, H-1’) appeared in the ^1^H-NMR spectrum of **2**. In its ^13^C-NMR spectrum, a set of signals at δ 18.0 (C-6’), 70.1 (C-5’), 72.3 (C-2’), 72.4 (C-3’), 74.0 (C-4’), and 101.4 (C-1’) also evidenced the presence of rhamnoside [39]. The observed HMBC correlations from H-2 to C-3, C-4, and C-6; from H-4 to C-6; from H-5 to C-3 and C-6; from H-7 to C-5, C-6, and C-1′; from H-1′ to C-3′ and C-5′, respectively, established the structure of **2** as 6-(α-rhamnosyloxymethyl)-3-pyridinol (Figure 2) and assigned the trivial name as viglutoside.

Viglutanone (**3**) was isolated as brown syrup, and the molecular formula was determined as C_9_H_10_O_5_ by a deprotonated molecular ion peak at *m*/*z* 197.0449 in the HR-ESI-MS analysis. The UV absorption maximum at 299 nm and IR absorption bands at 3493, 1703, and 1631 cm^−^^1^, along with the consideration of IHD value (5) proposed the presence of an α-pyranone basic structure with a carboxylic acid group. It was further evidenced by the proton signals for two vinyl singlets at δ 6.25 (1H, H-3) and 6.97 (1H, H-5) in ^1^H-NMR, and the carbon peaks for two vinyl groups at δ 110.4 (C-5), 114.8 (C-3), 156.4 (C-6), and 162.3 (C-4), and two carbonyls at δ 164.8 (C-2) and 165.8 (C-10), respectively. In addition, three methylene groups responsible for the propyloxyl substituent at δ 1.84 (2H, tt, *J* = 8.0, 6.4 Hz, H-8), 2.60 (2H, t, *J* = 8.0 Hz, H-7), and 3.60 (2H, t, *J* = 6.4 Hz, H-9) were observed in the ^1^H-NMR spectrum of **3**. The *^2^J*- and *^3^J*-HMBC correlations from H-3 to C-2 and C-7; from H-5 to C-3, C-6, C-7, and C-10; from H-7 to C-4; and from H-9 to C-7 and C-8 concluded the structure of **3** as the α-pyranone basic skeleton substituted with a propyloxyl fragment at C-4 and a carboxyl group at C-6 as shown (Figure 2).

**Figure 3:**
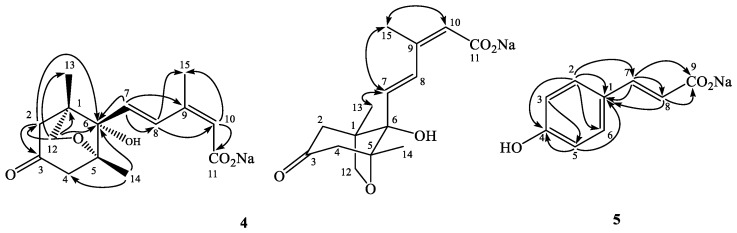
Key HMBC (→) and NOESY (↔) correlations of **4** and **5**.

In addition to new compounds **1**–**3**, two sodium salts **4** and **5** were also characterized from the methanol extract of *V. luteola*. Compound **4** was obtained as optically active white powder, and exhibited a protonated molecular at *m*/*z* 279.1234 in the HR-ESI-MS experiment. The ultraviolet (UV) absorption maxima at 249 nm and the IR absorption bands at 3424, 1713, and 1644 cm^−^^1^ suggested the occurrences of hydroxyl, carbonyl, and conjugated carbonyl groups in the molecule. The ^1^H-NMR spectrum of **4** exhibited signals for two trans olefinic protons at δ 6.20 (1H, d, *J* = 16.0 Hz, H-7) and 7.87 (1H, d, *J* = 16.0 Hz, H-8); one vinyl proton at δ 5.86 (1H, br s, H-10); three methyl singlets at δ 1.00 (3H, CH_3_-13), 1.22 (3H, CH_3_-14), and 1.95 (3H, CH_3_-15); and three methylene groups at δ 2.37 (1H, dd, *J* = 18.0, 2.4 Hz, H-2a), 2.45 (1H, dd, *J* = 18.0, 2.4 Hz, H-4a), 2.71 (1H, dd, *J* = 18.0, 2.4 Hz, H-2b), 2.81 (1H, d, *J* = 18.0 Hz, H-4b), 3.65 (1H, d, *J* = 7.2 Hz, H-12a), and 3.93 (1H, dd, *J* = 7.2, 2.8 Hz, H-12b). The ^13^C-NMR spectrum also displayed one carbonyl carbon at δ 211.3 (C-3); one carboxyl signal at δ 175.0 (C-11); three methyl carbons at δ 15.8 (C-13), 19.4 (C-14), and 20.5 (C-15); three methylene carbons at δ 53.2 (C-2), 54.0 (C-4), and 78.5 (C-12); three methines at δ 127.9 (C-10), 129.2 (C-7), and 134.1 (C-8); and four quaternary carbons at δ 49.0 (C-1), 82.8 (C-6), 87.8 (C-5), and 141.6 (C-9), respectively. The ^2^*J*- and ^3^*J*-HMBC correlations from H-2 to C-3, C-6; from H-7 to C-6, C-8, and C-9; from H-8 to C-10, and CH_3_-15; from H-10 to C-11 and CH_3_-15; from H-12 to C-1, C-2, C-6, and CH_3_-13; and from CH_3_-14 to C-4 and C-6, respectively, provided the possible planar structure as shown (Figure 3). Compared the spectral data of **4** with those of (−)-phaseic acid [40], the upfield shift of H-5 (δ 8.11 to 7.87) and ^13^C NMR signal of the carboxylate at δ 175.0 (C-11) further indicated the occurrence of sodium carboxylate. The presence of **4** as a sodium salt was evidenced by the inductively coupled plasma (ICP) MS analytical data, in which [Na^+^] was equal to 0.8 ppm in the 10 ppm sample. After acidification of **4** with 0.1 M HCl, the spectral data was changed to be the same as that of (−)-phaseic acid [40]. Furthermore, the NOE correlations of H-7 and CH_3_-13, H-7 and CH_3_-15, and H-10 and CH_3_-15, confirmed the relative stereochemical structure of **4** as sodium phaseate (Figure 3).

Compound **5** was purified as colorless powder and its molecular formula was characterized as C_9_H_7_O_3_ according to the HR-ESI-MS data. The UV absorption maxima at 282 nm and the IR absorption bands at 3403 and 1550 cm^−^^1^ suggested the occurrence of hydroxyl and carboxyl groups in the molecule. Only A_2_B_2_-coupled aromatic protons at δ 6.75 (2H, d, *J* = 8.8 Hz, H-3, 5) and 7.36 (2H, d, *J* = 8.8 Hz, H-2, 6), and two *trans* olefinic protons at δ 6.33 (1H, d, *J* = 15.6 Hz, H-8) and 7.33 (1H, d, *J* = 15.6 Hz, H-7) could be observed in its ^1^H-NMR spectrum. Compared the spectral data of **5** with those of *p*-coumaric acid [41], the upfield shift of H-7 (δ 7.60 to 7.33) and ^13^C NMR signal of the carboxylate at δ 176.4 (C-9) further indicated the occurrence of sodium carboxylate in **5**. The ^2^*J*- and ^3^*J*-HMBC correlations from H-2 to C-1, C-4, C-6, and C-7; from H-3 to C-5; from H-5 to C-1 and C-4; from H-7 to C-1, C-8, and C-9; and from H-8 to C-1 and C-9, respectively, evidenced the planar structure of **5** (Figure 3). The presence of **5** as a sodium salt was evidenced by the ICPMS analytical data, in which [Na^+^] was equal to 2 ppm in the 10 ppm sample. After acidification of **5** with 0.1 M HCl, the spectral data was changed to be the same as that of *p*-coumaric acid [41]. These data determined the structure of **5** as sodium *p*-coumarate. Although compound **5** was already reported in 2012 [42], the present research is the first report of **5** from the natural sources.

### 2.3. Anti-Inflammatory Activity

Most of the purified compounds were examined for their inhibitory activity against the superoxide anion and elastase release by human neutrophils in response to fMLP/CB [43] (Table S1), and only those compounds with IC_50_ values lower than 10 μM are listed in Table 2. The experimental data indicated **26**, **54**, **55**, **57**, **63**, **65**, **67**, and **70** displayed significant inhibition of superoxide anion generation with IC_50_ values ranged from 1.9 ± 0.2 to 9.3 ± 0.3 μM, as compared with the positive control LY294002 (IC_50_ 1.0 ± 0.2 μM). In addition, **55**, **57**, **63**, **65**, **66**, and **67** exhibited significant inhibition of elastase release with IC_50_ values ranged from 3.8 ± 0.1 to 7.7 ± 0.5 μM, as compared with the positive control LY294002 (IC_50_ 3.1 ± 0.7 μM). These experimental results indicated that flavonoids and isoflavonoids in *Vigna* species displayed significant chemoprevention potentials. According to the anti-inflammatory activity examinations performed in this study, the crude extract and purified constituents of *V. luteola* could be developed as new lead compounds or health food ingredients in the future.

## 3. Experimental Section

### 3.1. General

The spectroscopic data of the purified compounds including optical rotations ([α]_D_^25^) and UV and IR spectra were recorded on a Jasco P-2000 digital polarimeter (Jasco, Tokyo, Japan), a Hitachi U-0080D diode array spectrophotometer (Hitachi, Tokyo, Japan), and a Jasco FT/IR-4100 spectrophotometer (Jasco, Tokyo, Japan), respectively. The mass spectra were collected on a Shimadzu LC-MS 8040 spectrometer (Shimadzu, Kyoto, Japan). The HRMS data were obtained on a JMS-T100LP spectrometer (Jeol, Tokyo, Japan). ICPMS examination was performed on a Thermo-Element XR ICPMS spectrometer, and three elements (Na, K, and Ca) were examined for their concentration by comparison with the standard curves. ^1^H-, ^13^C-, and 2D NMR spectra were recorded on the Bruker AV-500 and Avance III-400 NMR spectrometers (Bruker, Billerica, MA, USA) using tetramethylsilane as the internal standard, and all NMR experiments were detected on standard pulse sequences and parameters with all chemical shifts reported in parts per million (ppm, δ). The deuterated solvents were purchased from Sigma-Aldrich (St. Louis, MO, USA). Other chemicals used in this study were provided by Merck KGaA (Darmstadt, Germany). Column chromatography was performed on silica gels in different mesh sizes (70–230 and 230–400 mesh, Kieselgel 60, Merck KGaA, Darmstadt, Germany). Thin-layer chromatography (TLC) was conducted on precoated Kieselgel 60 F 254 plates (Merck KGaA, Darmstadt, Germany). The spots on TLC were detected by UV light or spraying with 10 % (*v*/*v*) H_2_SO_4_ followed by heating at 110 °C for 10 min.

### 3.2. Plant Materials

The plant material *Vigna luteola* (Jacq.) Benth. was gathered in the side of Ching-Shui River, Nantou, Taiwan (March 2006), and it was authenticated by Prof. C. S. Kuoh (Department of Life Science, National Cheng Kung University, Tainan, Taiwan). A voucher specimen (PCKuo_2006002) was deposited in the herbarium of School of Pharmacy, National Cheng Kung University, Tainan, Taiwan.

### 3.3. Extraction and Isolation

The herbs of *V. luteola* (dried weight: 3.5 kg) were grounded and extracted with methanol (20 L) exhaustively under reflux (85 °C) for 8 hours, and the resulting liquid was concentrated in vacuo to give a dark brown syrup (640 g). The methanol extract was partitioned between chloroform and water to produce chloroform soluble layer (190 g) and water soluble layer (450 g), respectively.

The chloroform layer was subjected to a silica gel column eluted with a step gradient of *n*-hexane and acetone (100:1 to 1:1) to afford seven fractions (CF 1–7), as monitored by TLC. CF 3 was further column chromatographed on silica gel with a mixture of *n*-hexane and ethyl acetate (step gradient from 50:1 to 1:1) to afford fourteen subfractions (CF 3-1–3-14). CF 3-2 was purified by silica gel column chromatography (SiO_2_ CC) and the minor fraction was recrystallized from chloroform to give **18** (0.3 g). CF 3-3 was isolated by SiO_2_ CC to yield **16** (0.8 mg), **17** (1.1 mg), **40** (1.8 mg), a mixture of **19** and **20** (2.5 mg), and a mixture of **23** and **24** (2.2 mg), respectively. CF 3-5 was separated by SiO_2_ CC and further preparative thin-layer chromatography (pTLC) purification afforded compounds **6** (1.6 mg), **7** (4.3 mg), **37** (4.2 mg), **69** (0.6 mg), **8** (5.4 mg), **14** (1.2 mg), **55** (1.8 mg), and **63** (2.0 mg), respectively. CF 6 was further subjected on a silica gel column and eluted with a chloroform and methanol mixture (step gradient from 50:1 to 1:1) to produce thirteen subfractions (CF 6-1–6-13). A mixture of **21** and **22** (4.3 mg) was obtained from CF 6-7 by recrystallization of ethyl acetate. CF 6-8 was purified by SiO_2_ CC and the minor fraction was further isolated by pTLC with a solvent mixture of chloroform and acetone (10:1) to obtain **41** (1.0 mg). CF 7 was isolated on a SiO_2_ CC eluted with chloroform and methanol (step gradient from 50:1 to 1:1) to give twelve subfractions (CF 7-1–7-12). CF 7-3, 7-4, 7-5, and 7-8 were purified by SiO_2_ CC and the resulting minor fractions were subjected to pTLC to yield **68** (2.3 mg), **25** (1.9 mg) and **64** (1.5 mg), **10** (1.6 mg) and **43** (5.1 mg), and **36** (2.2 mg), respectively.

The water soluble layer was resolved on a Diaion HP-20 column and eluted with a step gradient mixture of water and methanol (10:0, 7:3, 5:5, 3:7, and 0:10) to result in sixteen fractions (WF 1–16). WF 1 was subjected to Diaion HP-20 CC eluted with the same program as mentioned above to obtain nine subfractions (WF 1-1–1-9). WF 1-3 was purified by pTLC with a solvent mixture of chloroform and methanol (50:1) to give **26** (3.1 mg) and **38** (1.5 mg). WF 1-4 was separated by SiO_2_ CC and further purified by pTLC to afford **65** (2.2 mg), **67** (6.0 mg), **35** (24.0 mg), and **54** (2.7 mg). WF 3 was purified by Sephadex LH-20 CC eluted with a step gradient mixture of water and methanol (10:0, 7:3, 5:5, 3:7, and 0:10) to produce thirteen subfractions (WF 3-1–3-13). WF 3-4 and 3-9 were separated by pTLC to yield **70** (1.3 mg), **48** (2.4 mg), and **56** (7.3 mg). WF 3-10 was purified by SiO_2_ CC eluted with a step gradient mixture of chloroform and methanol (50:1 to 1:1) to produce eight minor fractions (WF 3-10-1–3-10-8). WF 3-10-2 was isolated by reversed-phase HPLC with a Gemini 5u C18 column (250 × 4.6 mm, 5μm) eluted with a MeOH-H_2_O mixture (40:60, 0.4 mL/min) to yield **61** (2.7 mg) and **62** (3.5 mg). WF 5 was isolated by Diaion HP-20 CC to give six subfractions (WF 5-1 ~ 5-6). WF 5-4 was subjected on a Sephadex LH-20 column eluted with a mixture of water and methanol (10:0, 7:3, 5:5, 3:7, and 0:10), and then recrystallization of the resulting minor fractions afforded **66** (2.2 mg), **71** (1.1 mg), **4** (3.7 mg), **39** (1.6 mg), **44** (1.8 mg), and **57** (5.0 mg), respectively. WF 5-5 was purified by reversed-phase HPLC with a Gemini 5u C18 column (250 × 4.6 mm, 5μm) eluted with a MeOH-H_2_O mixture (20:80, 0.6 mL/min) to afford **52** (4.2 mg). WF 6 was subjected to Diaion HP-20 CC eluted with water and a step gradient of methanol (10:0 to 0:10) to afford six subfractions (WF 6-1–6-6). WF 6-2 and 6-6 were further purified by SiO_2_ CC and pTLC to obtain **5** (4.0 mg), **12** (2.6 mg), **49** (3.3 mg), **15** (3.5 mg), and **58** (0.4 g). WF 7 was purified by Diaion HP-20 CC eluted with water and a step gradient of methanol (10:0 to 0:10) to obtain seven subfractions (WF 7-1–7-7). WF 7-6 was separated by repeated Sephadex LH-20 CC eluted with a step gradient mixture of water and methanol (10:0 to 0:10) to yield **1** (2.8 mg), **9** (17.0 mg), **11** (5.2 mg), **47** (2.6 g), **59** (1.5 g), and **60** (0.5 g). WF 8 was purified by SiO_2_ CC eluted by chloroform and a step gradient with methanol and water (100:1:0.1 to 1:1:0.1) to obtain seven subfractions (WF 8-1–8-7). WF 8-4 was isolated by SiO_2_ CC and pTLC to give **45** (2.7 mg), **13** (1.1 mg), and **72** (1.9 mg). WF 10 was subjected to Sephadex LH-20 CC eluted with water and a step gradient of methanol (10:0 to 0:10) to obtain ten subfractions (WF 10-1–10-10). WF 10-3, 10-6, and 10-9 were further purified by pTLC to yield **42** (0.8 mg); **31** (4.2 mg), **46** (12.3 mg), and **50** (11.0 mg), and **51** (1.3 mg) and **53** (2.2 mg), respectively. WF 12 was subjected to Diaion HP-20 CC eluted with a mixture of water and methanol (step gradient from 10:0 to 0:10) to afford seven subfractions (WF 12-1–12-7). WF 12-3 was purified by Sephadex LH-20 CC and further resolved on pTLC to produce **30** (1.9 mg), **34** (2.1 mg), and **73** (1.4 mg). WF 12-6 was isolated by repeated SiO_2_ CC yield **2** (4.2 mg). WF 13 was resolved on a Sephadex LH-20 column eluted with water and methanol (step gradient from 10:0 to 0:10) to produce eight subfractions (WF 13-1–13-8). WF 13-3 was separated by SiO_2_ CC and pTLC to obtain **32** (1.6 mg) and **33** (40.0 mg). WF 13-7 was resolved on Sephadex LH-20 CC and the minor fraction was isolated by repeated SiO_2_ CC to result in **3** (6.3 mg), **27** (33.0 mg), and **28** (43.3 mg). WF 14 was subjected to SiO_2_ CC eluted with ethyl acetate and methanol (step gradient from 300:1 to 1:1) to give nine subfractions (WF 14-1–14-9). Compound **29** (7.5 mg) was obtained from the WF 14-7 by repeated SiO_2_ CC followed by recrystallization.

#### 3.3.1. Viglutin (1)

Colorless powder; [α]_D_^25^ + 36.9 (*c* 0.1, MeOH); IR (neat) ν_max_: 3416, 2928, 1647, 1580, 1383, 1045 cm^−1^; ESI-MS (*rel. int. %*) *m*/*z* 227 ([M + H]^+^, 100), 209 (43); HR-ESI-MS *m*/*z* 249.1460 ([M + Na]^+^) (Calcd for C_13_H_22_NaO_3_: 249.1467); ^1^H-NMR (CD_3_OD, 500 MHz) *δ* 5.78 (1H, dq, *J* = 15.5, 6.5 Hz, H-9), 5.67 (1H, ddd, *J* = 15.5, 8.5, 1.5 Hz, H-8), 4.18 (1H, d, *J* = 8.5 Hz, H-7), 3.81 (1H, m, H-13b), 3.80 (1H, dddd, *J* = 12.0, 7.5, 5.0, 3.5 Hz, H-3), 3.61 (1H, dd, *J* = 11.0, 7.0 Hz, H-13a), 2.44 (1H, m, H-5), 1.73 (1H, m, H-4b), 1.73 (3H, dd, *J* = 6.5, 1.5 Hz, CH_3_-10), 1.51 (1H, ddd, *J* = 12.0, 12.0, 11.5 Hz, H-4a), 1.36 (1H, ddd, *J* = 12.6, 5.0, 1.5 Hz, H-2b), 1.31 (1H, m, H-2a), 1.06 (3H, s, CH_3_-12), 0.96 (3H, s, CH_3_-11); ^13^C-NMR (CD_3_OD, 125 MHz) *δ* 132.1 (C-9), 129.6 (C-8), 84.4 (C-7), 81.2 (C-6), 69.7 (C-13), 68.5 (C-3), 46.8 (C-2), 43.7 (C-5), 37.7 (C-1), 31.6 (C-4), 25.9 (C-11), 24.0 (C-12), 18.2 (C-10).

#### 3.3.2. Viglutoside (2)

Yellowish amorphous powder; [α]_D_^25^ − 23.6 (*c* 0.3, MeOH); UV (MeOH) λ_max_ (log ε): 221 (3.78), 274 (3.38) nm; IR (neat) ν_max_: 3430, 2924, 1637 cm^−1^; ESI-MS (*rel. int. %*) *m*/*z* 272 ([M + H]^+^, 100); HR-ESI-MS *m/z* 294.0952 ([M + Na]^+^) (Calcd for C_12_H_17_NNaO_6_: 294.0954); ^1^H-NMR (CD_3_OD, 500 MHz) *δ* 8.03 (1H, d, *J* = 2.5 Hz, H-2), 7.32 (1H, d, *J* = 8.5 Hz, H-5), 7.21 (1H, dd, *J* = 8.5, 2.5 Hz, H-4), 4.78 (1H, d, *J* = 2.0 Hz, H-1’), 4.65 (1H, d, *J* = 12.0 Hz, H-7b), 4.51 (1H, d, *J* = 12.0 Hz, H-7a), 3.85 (1H, dd, *J* = 3.5, 2.0 Hz, H-2’), 3.67 (1H, dd, *J* = 9.5, 3.5 Hz, H-3’), 3.60 (1H, dd, *J* = 9.5, 6.0 Hz, H-5’), 3.38 (1H, dd, *J* = 9.5, 9.5 Hz, H-4’), 1.25 (1H, d, *J* = 6.0 Hz, H-6’); ^13^C-NMR (CD_3_OD, 125 MHz) *δ* 155.9 (C-3), 148.6 (C-6), 138.1 (C-2), 125.0 (C-4), 124.8 (C-5), 101.4 (C-1’), 74.0 (C-4’), 72.4 (C-3’), 72.3 (C-2’), 70.3 (C-7), 70.1 (C-5’), 18.0 (C-6’).

#### 3.3.3. Viglutanone (3)

Colorless syrup; UV (MeOH) λ_max_ (log ε): 299 (3.44) nm; IR (neat) ν_max_: 3493, 2953, 2133, 1703, 1631, 1387, 1054 cm^−1^; ESI-MS (*rel. int. %*) *m*/*z* 197 ([M-H]^−^, 100); HR-ESI-MS *m*/*z* 197.0449 ([M − H]^−^) (Calcd for C_9_H_9_O_5_: 197.0450); ^1^H-NMR (CD_3_OD, 400 MHz) *δ* 6.97 (1H, s, H-5), 6.25 (1H, s, H-3), 3.60 (1H, t, *J* = 6.4 Hz, H-9), 2.60 (1H, t, *J* = 8.0 Hz, H-7), 1.84 (1H, tt, *J* = 8.0, 6.4 Hz, H-8); ^13^C-NMR (CD_3_OD, 100 MHz) *δ* 165.8 (C-10), 164.8 (C-2), 162.3 (C-4), 156.4 (C-6), 114.8 (C-3), 110.4 (C-5), 61.7 (C-9), 32.6 (C-7), 32.1 (C-8).

#### 3.3.4. Sodium Phaseate (4)

Colorless powder; [α]_D_^25^ − 9.1 (*c* 0.5, MeOH); UV (MeOH) λ_max_ (log ε): 249 (3.55) nm; IR (neat) ν_max_: 3424, 2937, 1713, 1644, 1636, 1550, 1401, 1334, 1254 cm^−1^; HR-ESI-MS *m*/*z* 279.1234 ([M − Na]^−^) (Calcd for C_15_H_19_O_5_: 279.1233); ^1^H-NMR (CD_3_OD, 400 MHz) *δ* 7.87 (1H, d, *J* = 16.0 Hz, H-8), 6.20 (1H, d, *J* = 16.0 Hz, H-7), 5.86 (1H, br s, H-10), 3.93 (1H, dd, *J* = 7.2, 2.8 Hz, H-12b), 3.65 (1H, d, *J* = 7.2 Hz, H-12a), 2.81 (1H, d, *J* = 18.0 Hz, H-4b), 2.71 (1H, dd, *J* = 18.0, 2.4 Hz, H-2b), 2.45 (1H, dd, *J* = 18.0, 2.4 Hz, H-4a), 2.37 (1H, dd, *J* = 18.0, 2.4 Hz, H-2a), 1.95 (3H, s, CH_3_-15), 1.22 (3H, s, CH_3_-14), 1.00 (3H, s, CH_3_-13); ^13^C-NMR (CD_3_OD, 100 MHz) *δ* 211.3 (C-3), 175.0 (C-11), 141.6 (C-9), 134.1 (C-8), 129.2 (C-7), 127.9 (C-10), 87.8 (C-5), 82.8 (C-6), 78.5 (C-12), 54.0 (C-4), 53.2 (C-2), 49.0 (overlapped by MeOH, C-1), 20.5 (C-15), 19.4 (C-14), 15.8 (C-13).

#### 3.3.5. Sodium *p*-Coumarate (5)

Colorless powder; UV (MeOH) λ_max_ (log ε): 282 (3.83) nm; IR (neat) ν_max_: 3403, 2941, 1550, 1414, 1389, 1250, 1085 cm^−1^; ESI-MS (*rel. int. %*) *m*/*z* 163 ([M-Na]^-^, 100); HR-ESI-MS *m/z* 163.0396 ([M − Na]^−^) (Calcd for C_9_H_7_O_3_: 163.0395); ^1^H-NMR (CD_3_OD, 400 MHz) *δ* 7.36 (2H, d, *J* = 8.8 Hz, H-2, 6), 7.33 (1H, d, *J* = 15.6 Hz, H-7), 6.75 (2H, d, *J* = 8.8 Hz, H-3, 5), 6.33 (1H, d, *J* = 15.6 Hz, H-8); ^13^C-NMR (CD_3_OD, 100 MHz) *δ* 176.4 (C-9), 160.0 (C-4), 141.0 (C-7), 130.1 (C-2 and -6), 128.6 (C-1), 123.2 (C-8), 116.6 (C-3 and -5).

### 3.4. Anti-Inflammatory Bioactivity Examination

The assays of the generation of superoxide anion and elastase release inhibition examinations were determined as described previously [43]. The experimental details were provided in the Supplementary Materials.

## Figures and Tables

**Table 1:** Inhibitory percentages of crude extract and partial purified fractions of *V. luteola* on superoxide anion generation and elastase release by human neutrophils in response to *N*-formyl-l-methionyl-phenylalanine/cytochalasin B (fMLP/CB).

Samples	Inh % ^a^	
	Superoxide Anion Generation	Elastase Release
methanol extract	51.8 ± 6.8 ***	108.4 ± 6.9 ***
chloroform fraction	91.2 ± 3.8 ***	118.0 ± 5.0 ***
water fraction	18.3 ± 6.1 *	34.6 ± 3.6 ***

^a^ Percentage of inhibition (Inh %) at 10 μg/mL concentration. Results are presented as mean ± S.E.M. (n = 3). * *p* < 0.05, *** *p* < 0.001 compared with the control value (DMSO).

**Table 2:** Inhibitory effects of purified compounds on superoxide anion generation and elastase release by human neutrophils in response to fMLP/CB.

Compound	Superoxide Anion Generation		Elastase Release	
	IC_50_ (μM) ^a^	Inh % ^b^	IC_50_ (μM)	Inh %
**26**	6.1 ± 0.3	69.9 ± 4.4 ***	– ^c^	11.8 ± 2.1 **
**54**	4.5 ± 0.3	93.6 ± 3.3 ***	–	23.7 ± 1.1 ***
**55**	4.1 ± 0.2	99.0 ± 1.9 ***	3.8 ± 0.1	89.4 ± 4.5 ***
**57**	5.0 ± 0.4	88.4 ± 5.3 ***	4.7 ± 0.4	89.9 ± 2.2 ***
**63**	9.3 ± 0.3	52.5 ± 1.2 ***	4.9 ± 0.2	75.2 ± 3.2 ***
**65**	1.9 ± 0.2	89.3 ± 2.9 ***	6.4 ± 0.7	61.3 ± 4.7 ***
**66**	–	27.4 ± 7.5 *	7.7 ± 0.5	60.4 ± 2.3 ***
**67**	3.2 ± 0.1	100.0 ± 1.3 ***	4.1 ± 0.7	99.6 ± 7.6 ***
**70**	5.6 ± 0.9	85.2 ± 9.7 ***	–	46.5 ± 2.2 ***
**LY294002** ^d^	1.0 ± 0.2		3.1 ± 0.7	

Results are presented as mean ± SEM (n = 3). * *p* < 0.05, ** *p* < 0.01, *** *p* < 0.001 compared with the control (DMSO). ^a^ Concentration necessary for 50 % inhibition (IC_50_). ^b^ Percentage of inhibition (Inh %) at 10 μM concentration. ^c^ Not determined. ^d^ A phosphatidylinositol-3-kinase inhibitor was used as a positive control.

## Supplementary Materials

The following are available online, S1: Complete extraction and isolation procedures; S2: References of the known compounds; S3: Anti-inflammatory bioactivity experimental procedures; Table S1: Inhibitory effects of isolated compounds; Figures S1–37: NMR spectra of compounds **1**–**5**.
