# Supplementary material for: Chemical Constituents of Vigna luteola and Their Anti-inflammatory Bioactivity
Source: Molecules. 2019 Apr 8;24(7):1371. doi: 10.3390/molecules24071371 (PMC6479608; doi:10.3390/molecules24071371)
Supplement: Supplementary file 1 [file molecules-24-01371-s001.pdf]

Supplementary Materials

Chemical Constituents of *Vigna luteola* and Their Anti-inflammatory Bioactivity

Sio-Hong Lam [1,†], Yue-Chiun Li [2,†], Ping-Chung Kuo [1,*], Tsong-Long Hwang [3,4,5], Mei-Lin Yang [1], Chien-Chiao Wang [3], and Jason T.C. Tzen [2,*]

[1] School of Pharmacy, College of Medicine, National Cheng Kung University, Tainan 701, Taiwan; shlam@mail.ncku.edu.tw (S.-H.L); L3891104@nckualumni.org.tw (M.-L.Y.)

[2] Graduate Institute of Biotechnology, National Chung-Hsing University, Taichung 402, Taiwan; ycli0126@gmail.com (Y.-C.L.)

[3] Graduate Institute of Natural Products, College of Medicine, Chang Gung University, Taoyuan 333, Taiwan; htl@mail.cgu.edu.tw (T.-L.H.); D0501502@cgu.edu.tw (C.-C.W.)

[4] Research Center for Industry of Human Ecology, Research Center for Chinese Herbal Medicine, and Graduate Institute of Health Industry Technology, Chang Gung University of Science and Technology, Taoyuan 333, Taiwan

[5] Department of Anesthesiology, Chang Gung Memorial Hospital, Taoyuan 333, Taiwan

* Correspondence: z10502016@email.ncku.edu.tw (P.-C.K.), tctzen@dragon.nchu.edu.tw (J.T.-C.T.); Tel.: +886-6-2353535 # 6806 (P.-C.K.), +886-4-22840328 (J.T.-C.T.).

[†] Both authors contributed equally to this work.

Contents

S1. Complete extraction and isolation procedures.

S2. References of the known compounds.

S3. Anti-inflammatory bioactivity experimental procedures.

Table S1. Inhibitory effects of isolated compounds on superoxide anion generation and elastase release by human neutrophils in response to fMLP/CB.

Fig. S1. ^{1}H NMR spectrum of **1** (CD_3OD, 500 MHz).

Fig. S2. ^{13}C and DEPT NMR spectrum of **1** (CD_3OD, 125 MHz).

Fig. S3. COSY spectrum of **1** (CD_3OD, 500 MHz).

Fig. S4. HMQC spectrum of **1** (CD_3OD, 500 MHz).

Fig. S5. HMBC spectrum of **1** (CD_3OD, 500 MHz).

Fig. S6. NOESY spectrum of **1** (CD_3OD, 500 MHz).

Fig. S7. HRMS spectrum of **1**.

Fig. S8. ^{1}H NMR spectrum of **2** (CD_3OD, 500 MHz).

Fig. S9. ^{13}C and DEPT NMR spectrum of **2** (CD_3OD, 125 MHz).

Fig. S10. COSY spectrum of **2** (CD_3OD, 500 MHz).

Fig. S11. HMQC spectrum of **2** (CD_3OD, 500 MHz).

Fig. S12. HMBC spectrum of **2** (CD_3OD, 500 MHz).

Fig. S13. NOESY spectrum of **2** (CD_3OD, 500 MHz).

Fig. S14. MS/HRMS spectrum of **2**.

Fig. S15. ^{1}H NMR spectrum of **3** (CD_3OD, 400 MHz).

Fig. S16. ^{13}C and DEPT NMR spectrum of **3** (CD_3OD, 100 MHz).

Fig. S17. COSY spectrum of **3** (CD_3OD, 400 MHz).

Fig. S18. HMQC spectrum of **3** (CD_3OD, 400 MHz).

Fig. S19. HMBC spectrum of **3** (CD_3OD, 400 MHz).

Fig. S20. NOESY spectrum of **3** (CD_3OD, 400 MHz).

Fig. S21. MS/HRMS spectrum of **3**.

Fig. S22. ^{1}H NMR spectrum of **4** (CD_3OD, 400 MHz).

Fig. S23. ^{13}C and DEPT NMR spectrum of **4** (CD_3OD, 100 MHz).

Fig. S24. COSY spectrum of **4** (CD_3OD, 400 MHz).

Fig. S25. HMQC spectrum of **4** (CD_3OD, 400 MHz).

Fig. S26. HMBC spectrum of **4** (CD_3OD, 400 MHz).

Fig. S27. NOESY spectrum of **4** (CD_3OD, 400 MHz).

Fig. S28. ^1H NMR spectrum of **4** after acidification (CD_3OD, 500 MHz).

Fig. S29. MS/HRMS spectrum of **4**.

Fig. S30. ^1H NMR spectrum of **5** (CD_3OD, 400 MHz).

Fig. S31. ^{13}C and DEPT NMR spectrum of **5** (CD_3OD, 100 MHz).

Fig. S32. COSY spectrum of **5** (CD_3OD, 400 MHz).

Fig. S33. HMQC spectrum of **5** (CD_3OD, 400 MHz).

Fig. S34. HMBC spectrum of **5** (CD_3OD, 400 MHz).

Fig. S35. NOESY spectrum of **5** (CD_3OD, 400 MHz).

Fig. S36. ^1H NMR spectrum of **5** after acidification (CD_3OD, 500 MHz).

Fig. S37. MS/HRMS spectrum of **5**.

S1. Complete extraction and isolation procedures.

The herbs of *V. luteola* (dried weight 3.5 kg) were grounded and extracted with methanol (20 L) exhaustively under reflux (85 ℃) for 8 hours, and the resulting liquid was concentrated in vacuo to give a dark brown syrup (640 g). The methanol extract was partitioned between chloroform and water to produce chloroform soluble layer (190 g) and water soluble layer (450 g), respectively.

The chloroform layer was subjected to a silica gel column eluted with a step gradient of *n*-hexane and acetone (100:1 to 1:1) to afford seven fractions (CF 1 ~ 7) as monitored by TLC. CF 3 was further column chromatographed on silica gel with a mixture of *n*-hexane and ethyl acetate (step gradient from 50:1 to 1:1) to afford fourteen subfractions (CF 3-1 ~ 3-14). CF 3-2 was purified by silica gel column chromatography (SiO_2 CC) to yield nine minor fractions which eluted with a step gradient mixture of *n*-hexane and ethyl acetate (100:1 to 1:1). The minor fraction 3-2-6 was recrystallized from chloroform to give **18** (0.3 g). CF 3-3 was isolated by SiO_2 CC with a step gradient mixture of *n*-hexane and acetone eluent (50:1) to afford eleven minor fractions (CF 3-3-1 ~ 3-3-11). The CF 3-3-4 was purified by repeated SiO_2 CC eluted with *n*-hexane and acetone (step gradient 50:1 to 1:1) to yield **16** (0.8 mg), **17** (1.1 mg), and **40** (1.8 mg). CF 3-3-7 was purified by SiO_2 CC with a mixture of *n*-hexane and acetone (50:1) and further recrystallization of the resulting fractions afforded a mixture of **19** and **20** (2.5 mg), a mixture of **23** and **24** (2.2 mg), respectively. CF 3-5 was separated by SiO_2 CC eluted by chloroform/methanol (100:1 to 1:1) to afford fourteen minor fractions (3-5-1 ~ 3-5-14). Compounds **6** (1.6 mg) and **7** (4.3 mg) were yielded from CF 3-5-7 by preparative thin layer chromatography (pTLC) purification which developed with a solvent mixture of *n*-hexane and ethyl acetate (50:1). CF 3-5-10 was isolated by pTLC eluted with a solvent mixture of chloroform and methanol (100:1) to give **37** (4.2 mg) and **69** (0.6 mg). CF 3-5-11 was further separated by repeated SiO_2 CC eluted with a step gradient mixture of chloroform and methanol (50:1 to 1:1) to result in **8** (5.4 mg), **14** (1.2 mg), **55** (1.8 mg), and **63** (2.0 mg). Fraction 6 (CF 6) was further subjected on a silica gel column and eluted with a chloroform and methanol mixture (step gradient from 50:1 to 1:1) to produce thirteen subfractions (CF 6-1 ~ 6-13). A mixture of **21** and **22** (4.3 mg) was obtained from CF 6-7 by recrystallization of ethyl acetate. CF 6-8 was purified by SiO_2 CC with a mixture of benzene and ethyl acetate (30:1) to yield ten minor fractions (CF 6-8-1 ~ 6-8-10). CF 6-8-6 was further isolated by pTLC with a solvent mixture of chloroform and acetone (10:1) to obtain **41** (1.0 mg). CF 7 was isolated on a SiO_2

CC eluted with chloroform and methanol (step gradient from 50:1 to 1:1) to give twelve subfractions (CF 7-1 ~ 7-12). CF 7-3 was further purified by SiO_2 CC eluted with a mixture of chloroform and methanol (100:1) to obtain eight minor fractions (7-3-1 ~ 7-3-8). CF 7-3-5 was subjected to pTLC with a solvent mixture of chloroform and methanol (30:1) to afford **68** (2.3 mg). CF 7-4 was isolated with SiO_2 CC eluted with a mixture of chloroform and ethyl acetate (20:1) to produce ten minor fractions (CF 7-4-1 ~ 7-4-10). CF 7-4-3 was further purified by pTLC with a solvent mixture of *n*-hexane and ethyl acetate (20:1) to give **25** (1.9 mg) and **64** (1.5 mg). CF 7-5 was subjected to SiO_2 CC eluted with a mixture of chloroform and methanol (step gradient from 200:1 to 1:1) to obtain eleven minor fractions (CF 7-5-1 ~ 7-5-11). CF 7-5-5 was further purified by SiO_2 CC eluted with a mixture of chloroform and methanol (300:1) to produce twelve minor subfractions (CF 7-5-5-1 ~ 7-5-5-12). CF 7-5-5-6 was isolated by pTLC with a solvent mixture of chloroform and methanol (20:1) to yield **10** (1.6 mg) and **43** (5.1 mg). Compound **36** (2.2 mg) was purified from CF 7-8 by repeated SiO_2 CC with ethyl acetate and methanol eluent (100:1 to 1:1).

The water soluble layer was resolved on a Diaion HP-20 column and eluted with a step gradient mixture of water and methanol (10:0, 7:3, 5:5, 3:7, 0:10) to result in sixteen fractions (WF 1 ~ 16). WF 1 was subjected to Diaion HP-20 CC eluted with the same program as mentioned above to obtain nine subfractions (WF 1-1 ~ 1-9). WF 1-3 was purified by pTLC with a solvent mixture of chloroform and methanol (50:1) to give **26** (3.1 mg) and **38** (1.5 mg). WF 1-4 was separated by SiO_2 CC eluted with a step gradient mixture of chloroform and methanol to obtain nine minor fractions (WF 1-4-1 ~ 1-4-9). WF 1-4-2 was separated by pTLC eluted with a solvent mixture of chloroform and methanol (30:1) to give **65** (2.2 mg) and **67** (6.0 mg). Compound **35** (24.0 mg) and **54** (2.7 mg) were obtained from WF 1-4-6 by pTLC eluted with a solvent mixture of ethyl acetate and methanol (50:1). WF 3 was purified by Sephadex LH-20 CC eluted with a step gradient mixture of water and methanol (10:0, 7:3, 5:5, 3:7, 0:10) to produce thirteen subfractions (WF 3-1 ~ 3-13). WF 3-4 was separated by pTLC eluted with a solvent mixture of chloroform and methanol (10:1) to yield **70** (1.3 mg). WF 3-9 was resolved on SiO_2 CC and separated by a solvent mixture of ethyl acetate and methanol (30:1) to obtain **48** (2.4 mg) and **56** (7.3 mg). WF 3-10 was purified by SiO_2 CC eluted with a step gradient mixture of chloroform and methanol (50:1 to 1:1) to produce eight minor fractions (WF 3-10-1 ~ 3-10-8). WF 3-10-2 was isolated by reversed-phase HPLC with a Gemini 5u C18 column (250 × 4.6 mm, 5μm) eluted with a MeOH-H_2O mixture (40:60, 0.4 mL/min) to yield **61** (2.7 mg) and **62** (3.5 mg). WF 5 was isolated by Diaion HP-20 CC with the same

program as mentioned above to give six subfractions (WF 5-1 ~ 5-6). WF 5-4 was subjected on a Sephadex LH-20 column eluted with a mixture of water and methanol (10:0, 7:3, 5:5, 3:7, 0:10) to afford nine minor fractions (WF 5-4-1 ~ 5-4-9). WF 5-4-2 was separated by repeated SiO_2 CC with a mixture of chloroform and methanol (step gradient from 100:1 to 1:1), and then recrystallization of the resulting fractions afforded **66** (2.2 mg) and **71** (1.1 mg). WF 5-4-4 was further isolated by repeated Sephadex LH-20 CC eluted with a mixture of water and methanol (10:0, 7:3, 5:5, 3:7, 0:10) resulting in **4** (3.7 mg), **39** (1.6 mg), **44** (1.8 mg), and **57** (5.0 mg), respectively. WF 5-5 was purified by reversed-phase HPLC with a Gemini 5u C18 column (250 × 4.6 mm, 5μm) eluted with a MeOH-H_2O mixture (20:80, 0.6 mL/min) to afford **52** (4.2 mg). WF 6 was subjected to Diaion HP-20 CC eluted with water and a step gradient of methanol (10:0 to 0:10) to afford six subfractions (WF 6-1 ~ 6-6). WF 6-2 was further purified by SiO_2 CC with a mixture of chloroform and methanol (50:1) to produce five minor fractions (WF 6-2-1 ~ 6-2-5). WF 6-2-4 was separated by pTLC with a solvent mixture of chloroform, methanol and water (10:1:0.1) to obtain **5** (4.0 mg), **12** (2.6 mg) and **49** (3.3 mg). WF 6-6 was resolved on SiO_2 CC eluted with ethyl acetate and methanol (300:1) to give seven minor fractions (WF 6-6-1 ~ 6-6-7). WF 6-6-7 was isolated by pTLC eluted with chloroform and methanol (10:1) to yield **15** (3.5 mg) and **58** (0.4 g). WF 7 was purified by Diaion HP-20 CC eluted with water and a step gradient of methanol (10:0 to 0:10) to obtain seven subfractions (WF 7-1 ~ 7-7). WF 7-6 was separated by repeated Sephadex LH-20 CC eluted with a step gradient mixture of water and methanol (10:0 to 0:10) to yield **1** (2.8 mg), **9** (17.0 mg), **11** (5.2 mg), **47** (2.6 g), **59** (1.5 g), and **60** (0.5 g). WF 8 was purified by SiO_2 CC eluted by chloroform and a step gradient with methanol and water (100:1:0.1 to 1:1:0.1) to obtain seven subfractions (WF 8-1 ~ 8-7). WF 8-4 was isolated by SiO_2 CC with a solvent mixture of ethyl acetate and methanol (20:1) to give five minor fractions (WF 8-4-1 ~ 8-4-5). WF 8-4-3 was recrystallized with ethyl acetate to give **45** (2.7 mg). WF 8-4-5 was resolved on pTLC and purified with a solvent mixture of chloroform, methanol and water (6:1:0.1) to yield **13** (1.1 mg) and **72** (1.9 mg). Fraction 10 (WF 10) was subjected to Sephadex LH-20 CC eluted with water and a step gradient of methanol (10:0 to 0:10) to obtain ten subfractions (WF 10-1 ~ 10-10). WF 10-3 was purified by pTLC with a solvent mixture of chloroform and ethyl acetate (3:1) to give **42** (0.8 mg). WF 10-6 was isolated by SiO_2 CC eluted with ethyl acetate, methanol and water (50:1:0.1) to produce seven minor fractions (WF 10-6-1 ~ 10-6-7). WF 10-6-6 was purified by repeated SiO_2 CC eluted with a mixture of ethyl acetate and methanol (step gradient from 50:1 to 5:1) to yield **31** (4.2 mg), **46** (12.3 mg), and **50** (11.0 mg). WF 10-9 was separated by repeated SiO_2

CC eluted with mixture of chloroform and methanol, and then recrystallization of the resulting minor fractions to give **51** (1.3 mg) and **53** (2.2 mg). WF 12 was subjected to Diaion HP-20 CC eluted with a mixture of water and methanol (step gradient from 10:0 to 0:10) to afford seven subfractions (WF 12-1 ~ 12-7). WF 12-3 was purified by Sephadex LH-20 CC eluted with water and methanol (step gradient from 10:0 to 0:10) to give six minor fractions (WF 12-3-1 ~ 12-3-6). Compounds **30** (1.9 mg), **34** (2.1 mg) and **73** (1.4 mg) were obtained from WF 12-3-3 by resolving on pTLC with a solvent mixture of chloroform, methanol and water (10:1:01). WF 12-6 was isolated by repeated SiO_2 CC eluted with a mixture of chloroform, methanol and water (30:1:01) to yield **2** (4.2 mg). WF 13 was resolved on a Sephadex LH-20 column eluted with water and methanol (step gradient from 10:0 to 0:10) to produce eight subfractions (WF 13-1 ~ 13-8). WF 13-3 was separated by SiO_2 CC eluted with a step gradient mixture of chloroform and methanol to give seven minor fractions (WF 13-3-1 ~ 13-3-7). WF 13-3-3 was further purified by pTLC separated with a solvent mixture of chloroform, methanol and water (5:1:0.1) to obtain **32** (1.6 mg) and **33** (40.0 mg). WF 13-7 was resolved on Sephadex LH-20 CC eluted with a mixture of water and methanol (step gradient from 10:0 to 0:10) to yield eight minor fractions (WF 13-7-1 ~ 13-7-8). WF 13-7-7 was isolated by repeated SiO_2 CC eluted with a step gradient mixture of ethyl acetate, methanol and water (30:1:0.1 to 1:1:0.1) to result in **3** (6.3 mg), **27** (33.0 mg), and **28** (43.3 mg). WF 14 was subjected to SiO_2 CC eluted with ethyl acetate and methanol (step gradient from 300:1 to 1:1) to give nine subfractions (WF 14-1 ~ 14-9). Compound **29** (7.5 mg) was obtained from the WF 14-7 by repeated SiO_2 CC eluted with a solvent mixture of chloroform and methanol (50:1) followed by recrystallization.

S2. References of the known compounds.

loliolide (**6**): He, Z.; Zhang, A.; Ding, L.; Lei, X.; Sun, J.; Zhang, L. Chemical composition of the green alga *Codium divaricatum* Holmes. *Fitoterapia* **2010**, 81, 1125-1128.

isololiolide (**7**): Kimura, J.; Maki, N. New loliolide derivatives from the brown alga *Undaria pinnatifida*. *J. Nat. Prod.* **2002**, 65, 57-58.

blumenol A (**8**): Ito, N.; Etoh, T.; Hagiwara, H.; Kato, M. Novel synthesis of degradation products of carotenoids, megastigmatrienone analogues and blumenol-A. *J. Chem. Soc., Perkin Trans. 1* **1997**, 1, 1571-1579.

kiwiionol (**9**): Takeda, Y.; Okada, Y.; Masuda, T.; Hirata, E.; Shinzato, T.; Takushi, A.; Yu, Q.; Otsuka, H. New megastigmane and tetraketide from the leaves of *Euscaphis japonica*. *Chem. Pharm. Bull.* **2000**, 48, 752-754.

(+)-(*S*)-dehydrovomifoliol (**10**): Xiao, W. L.; Chen, W. H.; Zhang, J. Y.; Song, X. P.; Chen, G. Y.; Han, C. R. Ionone-type sesquiterpenoids from the stems of *Ficus pumila*. *Chem. Nat. Compd.* **2016**, 52, 531-533.

(6*S*,9*R*)-roseoside (**11**): Mamadalieva, N. Z.; Sharopov, F.; Girault, J. P.; Wink, M.; Lafont, R. Phytochemical analysis and bioactivity of the aerial parts of *Abutilon theophrasti* (Malvaceae), a medicinal weed. *Nat. Prod. Res.* **2014**, 28, 1777-1779.

(3*S*,5*R*,6*S*,7*E*,9*R*)-megastigman-7-en-3,6,9-triol (**12**): Kawakami, S.; Matsunami, K.; Otsuka, H.; Shinzato, T.; Takeda, Y. Crotonionosides A-G: Megastigmane glycosides from leaves of *Croton cascarilloides* Räuschel. *Phytochemistry* **2011**, 72, 147-153.

(3*S*,5*R*,6*R*,7*E*)-3,5,6-trihydroxymega-stigman-7-en-9-one (**13**): Broom, S. J.; Ede, R. M.; Wilkins, A. L. Synthesis of (±)-*E*-(1,2,4-trihydroxy-2,6,6-tirmethylcyclohexyl)-but-3-en-2-one: A novel degraded carotenoid isolated from New Zealand thyme (*Thymus vulgaris*) honey. *Tetrahedron Lett.* **1992**, 33, 3197-3200.

abscisic acid (**14**): Smith, T. R.; Clark, A. J.; Clarkson, G. J.; Taylor, P. C.; Marsh, A. Concise enantioselective synthesis of abscisic aicd and a new analogue. *Org. Biomol. Chem.* **2006**, 4, 4186-4192.

machilusoxide A (**15**): Kuo, Y. Z. Studies on the stem constituents of *Machilus philippinensis* Merr., Master Thesis, National Cheng Kung University, Tainan, **2007**.

3(17)-phytene-1,2-diol (**16**): Rodríguez, A. D.; Acosta, A. L. New cembranoid diterpenes and a geranylgeraniol derivative from the common Caribbean sea whip *Eunicea succinea*. *J. Nat. Prod.* **1997**, 60, 1134-1138.

lupeol (**17**): Fotie, J.; Bohle, D. S.; Leimanis, M. L.; Georges, E.; Rukunga, G.; Nkengfack, A. E. Lupeol long-chain fatty acid esters with antimalarial activity from *Holarrhena floribunda*. *J. Nat. Prod.* **2006**, 69, 62-67.

simiarenol (**18**): Heupel, R. C. Varietal similarities and differences in the polycyclic isopentenoid composition of sorghum. *Phytochemistry* **1984**, 24, 2929-2937.

mixture of *β*-sitosterol (**19**) and stigmasterol (**20**): Xu, S.; Shang, M. Y.; Liu, G. X.; Xu, F.; Wang, X.; Shou, C. C.; Cai, S. Q. Chemical constituents from the rhizomes of *Smilax glabra* and their antimicrobial activity. *Molecules* **2013**, 18, 5265-5287.

mixture of *β*-sitosteryl-3-*O*-*β*-D-glucoside (**21**) and stigmasteryl-3-*O*-*β*-D-glucoside (**22**), mixture of stigmast-4-en-3-one (**23**) and stigmast-4,22-dien-3-one (**24**), kaempferol (**54**): Chang, Y. C.; Chang, F. R.; Wu, Y. C. The constituents of *Lindera glauca*. *J. Chin. Chem. Soc.* **2000**, 47, 373-380.

ergosterol peroxide (**25**): Fangkrathok, N.; Sripanidkulchai, B.; Umehara, K.; Noguchi, H. Bioactive ergostanoids and a new polyhydroxyoctane from *Lentinus polychrous* mycelia and their inhibitory effects on E2-enhanced cell proliferation of T47D cells. *Nat. Prod. Res.* **2013**, 27, 1611-1619.

(+)-pinoresinol (**26**): Gabaston, J.; Richard, T.; Cluzet, S.; Pinto, A. P.; Dufour, M. C.; Corio-Costet, M. F.; Mérillon, J. M. *Pinus pinaster* knot: A source of polyphenols against *Plasmopara viticola*. *J. Agric. Food Chem.* **2017**, 65, 8884-8891.

uracil (**27**), 5,7,4'-trihydroxyisoflavone (**65**): Wei, J.; Zhang, X. Y.; Deng, S.; Cao, L.; Xue, Q. H.; Gao, J. M. *α*-Glucosidase inhibitors and phytotoxins from *Streptomyces xanthophaeus*. *Nat. Prod. Res.* **2017**, 31, 2062-2066.

uridine (**28**): Downey, A. M.; Richter, C.; Pohl, R.; Mahrwald, R.; Hocek, M. Direct one-pot synthesis of nucleosides from unprotected or 5-*O*-monoprotected D-ribose. *Org. Lett.* **2015**, 17, 4604-4607.

6-hydroxymethyl-3-pyridinol (**29**): Xie, G.; Qin, X.; Chen, Y.; Wen, R.; Wu, S.; Qin, M. Alkaloids from the rhizomes of *Iris germanica*. *Chem. Nat. Compd.* **2017**, 53, 196-198.

thymine (**30**): Gustavsson, T.; Sarkar, N.; Bányász, A.; Markovitsi, D.; Improta, R. Solvent effects on the steady-state absorption and fluorescence spectra of uracil, thymine and 5-fluorouracil. *Photochem. Photobiol.* **2007**, 83, 595-599.

adenine (**31**): Lenz, R.; Giese, B. Studies on the mechanism of ribonucleotide reductases. *J. Am. Chem. Soc.* **1997**, 119, 2784-2794.

nicotinamide (**32**): Kumar, S.; Das, P. Solid-supported ruthenium(0): an efficient heterogeneous catalyst for hydration of nitriles to amides under microwave irradiation. *New J.Chem.* **2013**, 37, 2987-2990.

potassium nicotinate (**33**): Koczoń, P.; Piekut, J.; Borawska, M.; Świsłocka, R.; Lewandowski, W. The relationship between chemical structure and antimicrobial activity of selected nicotinates, *p*-iodobenzoates, picolinates and isonicotinates. *Spectrochim. Acta A Mol. Biomol. Spectrosc.* **2005**, 61, 1917-1922.

6-hydroxynicotinic acid (**34**): Schippers, N.; Schwack, W. Synthesis of the 15N-labelled insecticide imidacloprid. *J. Label Compd. Radiopharm.* **2006**, 49, 305-310.

p-hydroxybenzoic acid (**35**): Xin, X. L.; Aisa, H. A.; Wang, H. Q. Flavonoids and phenolic compounds from seeds of the chinese plant *Nigella glandulifera*. *Chem. Nat. Compd.* **2008**, 44, 368-369.

methylparaben (**36**): Chen, C. Y.; Chang, F. R.; Teng, C. M.; Wu, Y. C. Cheritamine, a new N-fatty acyl tryptamine and other constituents from the stems of *Annona cherimola*. *J. Chin. Chem. Soc.* **1999**, 46, 77-86.

trans-methyl *p*-coumarate (**37**): Gopalakrishnan, S.; Subbarao, G. V.; Nakahara, K.; Yoshihashi, T.; Ito, O.; Maeda, I.; Ono, H.; Yoshida, M. Nitrification inhibitors from the root tissues of *Brachiaria humidicola*, a tropical grass. *J. Agric. Food Chem.* **2007**, 55, 1385-1388.

(*E*)-methyl ferulate (**38**), *trans*-isoferulic acid (**39**): Ahmad, I.; Chaudhary, B. A.; Janbaz, K. H.; Uzair, M.; Ashraf, M. Urease inhibitors and antioxidants from *Vernonia cinerascens*. *J. Chem. Soc. Pak.* **2011**, 33, 114-117.

trans-ferulic acid (**40**): Prachayasittikul, S.; Suphapong, S.; Worachartcheewan, A.; Lawung, R.; Ruchirawat, S.; Prachayasittikul, V. Bioactive metabolites from *Spilanthes acmella* Merr. *Molecules* **2009**, 14, 850-867.

isovanillic acid (**41**): Kobayashi, S.; Ozawa, T.; Imagawa, H. Dehydrochorismic acid from *Pinus densiflora* pollen. *Agric. Biol. Chem.* **1982**, 46, 845-847.

methyl vanillate (**42**): Martic, S.; Brennan, J. D.; Brook, M. A.; Ackloo, S.; Nagy, N. Towards the development of a covalently tethered MALDI system-a study of allyl-modified MALDI matrixes. *Can. J. Chem.* **2007**, 85, 66-76.

vanillic acid (**43**), syringic acid (**45**): Nishanbaev, S. Z.; Bobakulov, K. M.; Abdullaev, N. D.; Sham'yanov, I. D. Phenolcarboxylic acids from *Quercus robur* growing in Uzbekistan. *Chem. Nat. Compd.* **2015**, 51, 537-539.

4-hydroxybenzaldehyde (**44**): Tian, M. Q.; Wu, Q. L.; Wang, X.; Zhang, K. Q.; Li, G. H. A new compound from *Stereum insigne* CGMCC5.57. *Nat. Prod. Res.* **2017**, 31, 932-937.

gentisic acid (**46**): Tan, D.; Yan, Q.; Kang, H. Chemical constituents from *Blumea balsamifera*. *Chem. Nat. Compd.* **2013**, 48, 1072-1073.

sodium salicylate (**47**): Rao, U. R. K.; Manohar, C.; Valaulikar, B. S.; Iyer, R. M. Micellar chain model for the origin of the visoelasticity in dilute surfactant solutions. *J. Phys. Chem.* **1987**, 91, 3286-3291.

benzoic acid (**48**): Laurent, P.; Lebrun, B.; Braekman, J. C.; Daloze, D.; Pasteels, J. M. Biosynthetic studies on adaline and adalinine, two alkaloids from ladybird beetles (Coleopteral: Coccinellidae). *Tetrahedron* **2001**, 57, 3403-3412.

methyl 2-*O*-*β*-D-glucopyranosylsalicylate (**49**): Ushiyama, M.; Furuya, T. Glycosylation of phenolic compounds by root culture of *Panax ginseng*. *Phytochemistry* **1989**, 28, 3009-3013.

protocatechuic acid (**50**): Abdullah, N. H.; Salim, F.; Ahmad, R. Chemical constituents of *Malaysian U. cordata* var. *ferruginea* and their *in vitro α*-glucosidase inhibitory activities. *Molecules* **2016**, 21, 525.

phenylacetic acid (**51**): Gachet, M. S.; Kunert, O.; Kaiser, M.; Brun, R.; Muñoz, R. A.; Bauer, R.; Schühly, W. Jacaranone-derived glucosidic esters from *Jacaranda glabra* and their activity against *Plasmodium falciparum*. *J. Nat. Prod.* **2010**, 73, 553-556.

benzylalcohol *β*-D-glucopyranosyl(1→6)-*β*-D-glucopyranoside (**52**): De Rosa, S.; De Giulio, A.; Tommonaro, G. Aliphatic and aromatic glycosides from the cell cultures of *Lycopersicon esculentum*. *Phytochemistry* **1996**, 42, 1031-1034.

gentisic acid 5-*O*-*β*-D-xylopyranoside (**53**): Fayos, J.; Bellés, J. M.; López-Gresa, M. P.; Primo, J.; Conejero, V. Induction of gentisic acid 5-*O*-*β*-D-xylopyranoside in tomato and cucumber plants infected by different pathogens. *Phytochemistry* **2006**, 67, 142-148.

liquiritigenin (**55**): Kavtaradze, N. S.; Alaniya, M. D.; Mshvildadze, V. D.; Skhirtladze, A. V.; Lavoie, S.; Pichette, A. Flavonoids from *Astragalus microcephalus*. *Chem. Nat. Compd.* **2011**, 46, 971-973.

kaempferol 3-*O*-[*α*-L-rhamnopyranosyl(1→6)]-*β*-D-galactopyranoside (**56**): Rastrelli, L.; Saturnino, P.; Schettino, O.; Dini, A. Studies on the constituents of *Chenopodium*

pallidicaule (Cañihua) seeds. Isolation and characterization of two new flavonol glycosides. *J. Agric. Food Chem.* **1995**, 43, 2020-2024.

chrysoeriol (**57**): Noreen, H.; Farman, M.; McCullagh, J. S. O. Bioassay-guided isolation of cytotoxic flavonoids from aerial parts of *Coronopus didymus*. *J. Ethnopharmacol.* **2016**, 194, 971-980.

astragalin (**58**): Luyen, B. T. L.; Tai, B. H.; Thao, N. P.; Eun, K. J.; Cha, J. Y.; Xin, M. J.; Lee, Y. M.; Kim, Y. H. Anti-inflammatory components of *Euphorbia humifusa* Willd. *Bioorg. Med. Chem. Lett.* **2014**, 24, 1895-1900.

kaempferol-3-*O*-sophoroside (**59**): Chang, Y.; Zhang, P.; Zhang, X.; Chen, J.; Rausch, W. D.; Gula, A.; Bao, B. Cytotoxic activities of flavonoids from a traditional Mongolian medicinal herb *Clematis aethusifolia* Turcz. *Nat. Prod. Res.* **2017**, 31, 1223-1227.

kaempferol 3-*O*-sophoroside-7-*O*-rhamnoside (**60**): Kite, G. C.; Veitch, N. C.; Boalch, M. E.; Lewis, G. P.; Leon, C. J.; Simmonds, M. S. J. Flavonol tetraglycosides from fruits of *Styphnolobium japonicum* (Leguminosae) and the authentication of Fructus Sophorae and Flos Sophorae. *Phytochemistry* **2009**, 70, 785-794.

robinin (**61**): Asres, K.; Eder, U.; Bucar, F. Studies on the antiinflammatory activity of extracts and compounds from the leaves of *Melilotus elegans*. *Ethiop. Pharm. J.* **2000**, 18, 15-24.

isorhamnetin 3-*O*-[*α*-L-rhamnopyranosyl(1→6)]-*β*-D-galactopyranoside 7-*O*-*α*-L-rhamnopyranoside (**62**): Andersen, W. K.; Omar, A. A.; Christensen, S. B. Isorhamnetin3-(2,6-dirhamnosylgalactoside)-7-rhamnoside and 3-(6-rhamnosylgalactoside)-7-rhamnoside from *Rhazya stricta*. *Phytochemistry* **1986**, 26, 291-294.

daidzein (**63**): Biegasiewicz, K. F.; Denism, J. D. S.; Carroll, V. M.; Priefer, R. An efficient synthesis of daidzein dimethyldaidzein and isoformononetin. *Tetrahedron Lett.* **2010**, 51, 4408-4410.

8-*O*-methylretusin (**64**): Puebla, P.; Oshima-Franco, Y.; Franco, L. M.; Dos Santos, M. G.; da Silva, R. V.; Rubem-Mauro, L.; Feliciano, A. S. Chemical constituents of the bark of *Dipteryx alata* Vogel, an active species against *Bothrops jararacussu* Venom. *Molecules* **2010**, 15, 8193-8204.

7,2',4'-trihydroxyisoflavone (**66**): Woodward, M. D. Phaseollin formation and metabolism in *Phaseolus vulgaris*. *Phytochemistry* **1980**, 19, 921-927.

tectorigenin (**67**): Kim, J. M.; Ko, R. K.; Jung, D. S.; Kim, S. S.; Lee, N. H. Tyrosinase

inhibitory constituents from the stems of *Maackia fauriei*. *Phytother. Res.* **2010**, 24, 70-75.

afromosin (**68**): Nechepurenko, I. V.; Komarova, N. I.; Kuzovkina, I. N.; Vdovitchenko, M. Y.; Polovinka, M. P.; Salakhutdinov, N. F. Isolation and identification of 4',6-dimethoxy-7-hydroxyisoflavone from roots of *Hedysarum theinum* cultivated in vitro. *Chem. Nat. Compd.* **2009**, 45, 420-421.

3'-methoxydaidzein (**69**): Yahara, S.; Ogata, T.; Saijo, R.; Konishi, R.; Yamahara, J.; Miyahara, K.; Nohara, T. Isoflavan and related compounds from *Dalbergia odorifera*. I. *Chem. Pharm. Bull.* **1989**, 37, 979-987.

7,4'-dihydroxy-8-methoxyisoflavone (**70**): Shi, H. M.; Huang, Z. Q.; Wen, J.; Tu, P. F. A new isoflavone from *Abrus mollis*. *Chin. J. Nat. Med.* **2006**, 4, 30-31.

2-furanoic acid (**71**): Preobrazhenskaya, M. N.; Rozhkov, I. I.; Lazhko, E. I.; Yudina, L. N.; Korolev, A. M. Reaction of vanilmandelic acid and 4-hydroxybenzyl alcohol derivatives with L-ascorbic acid. *Tetrahedron* **1997**, 53, 6971-6976.

maltol glucoside (**72**): Adams, C. J.; Grainger, M. N. C.; Manley-Harris, M. Isolation of maltol glucoside from the floral nectar of New Zealand mānuka (*Leptospermum scoparium*). *Food Chem.* **2015**, 174, 306-309.

sodium 5-hydroxymethylfuran-2-carboxylate (**73**): Subbiah, S.; Simeonov, S. P.; Esperança, J. M. S. S.; Rebelo, L. P. N.; Afonso, C. A. M. Direct transformation of 5-hydroxymethylfurfural to the building blocks 2,5-dihydroxymethylfurfural (DHMF) and 5-hydroxymethyl furanoic acid (HMFA) *via* Cannizzaro reaction. *Green Chem.* **2013**, 15, 2849-2853.

S3. Anti-inflammatory bioactivity experimental procedures.

S3.1 Preparation of Human Neutrophils

A study involving human neutrophils was approved by the Institutional Review Board at Chang Gung Memorial Hospital, Taoyuan, Taiwan, and was conducted according to the Declaration of Helsinki (2013). The written informed consent was obtained from each healthy donor before blood was drawn. Blood was drawn from healthy human donors (20–30 years old) by venipuncture into heparin-coated vacutainer tubes, using a protocol approved by the Institutional Review Board at Chang Gung Memorial Hospital (No. 201601788A3). Blood samples were mixed gently with an equal volume of 3 % dextran solution. Neutrophils were isolated with a standard method of dextran sedimentation prior to centrifugation in a Ficoll Hypaque gradient and hypotonic lysis of erythrocytes. The leukocyte-rich plasma was collected after sedimentation of the red cells for 30 min at room temperature, and was transferred to 20 mL Ficoll solution (1.077 g/mL) and spun down at 400 g for 40 min at 20 ℃. The granulocyte/ erythrocyte pellets were resuspended in ice-cold 0.2 % NaCl to lyse erythrocytes. After 30 sec, the same volume of 1.6 % NaCl solution was added to reconstitute the isotonic condition. Purified neutrophils were pelleted and then resuspended in a calcium (Ca^{2+})-free Hank's balanced salt solution (HBSS) buffer at pH 7.4, and were maintained at 4 ℃ before use.

S3.2 Measurement of Superoxide Anion Generation

The assay of the generation of superoxide anion was based on the SOD-inhibitable reduction of ferricytochrome c. In brief, after supplementation with 0.6 μg/mL ferricytochrome c and 1 mM Ca^{2+}, neutrophils (6×10^5 cells/mL) were equilibrated at 37 ℃ for 2 min and incubated with drugs or an equal volume of vehicle (0.1 % DMSO, negative control) for 5 min. Cells were activated with 100 nM fMLP during the preincubation of 1 μg/mL cytochalasin B (fMLP/CB) for 3 min. Changes in the absorbance with a reduction in ferricytochrome c at 550 nm were continuously monitored in a double-beam, six-cell positioner spectrophotometer with constant stirring (Hitachi U-3010, Tokyo, Japan). Calculations were based on differences in the reactions with and without SOD (100 U/mL) divided by the extinction coefficient for the reduction of ferricytochrome c (ε = 21.1/mM/10 mm).

S3.3 Measurement of Elastase Release

Degranulation of azurophilic granules was determined by elastase release as described previously. Experiments were performed using MeO-Suc-Ala-Ala-Pro-Val-*p*-nitroanilide as the elastase substrate. Briefly, after supplement-ation with MeO-Suc-Ala-Ala-Pro-Val-*p*-nitroanilide (100 μM), neutrophils (6 × 10^5/mL) were equilibrated at 37 ℃ for 2 min and incubated with test compounds or an equal volume of vehicle (0.1 % DMSO, negative control) for 5 min. Cells were activated by 100 nM fMLP and 0.5 μg/mL cytochalasin B, and changes in absorbance at 405 nm were continuously monitored to assay elastase release. The results were expressed as the percent of elastase release in the fMLP/CB-activated, drug-free control system.

S3.4 Statistical Analysis

All the experiments were performed in triplicate. Results were expressed as means ± S.E.M. Calculations of 50 % inhibitory concentrations (IC_{50}) were computer-assisted (PHARM/PCS v.4.2). Statistical comparisons were made between groups using the Student's t test. Values of *p* less than 0.05 were considered to be statistically significant, and * $p < 0.05$, ** $p < 0.01$, *** $p < 0.001$, respectively.

Table S1. Inhibitory effects of isolated compounds on superoxide anion generation and elastase release by human neutrophils in response to fMLP/CB.

compound	superoxide anion generation		elastase release	
	IC_{50} (μM) [a]	Inh % [b]	IC_{50} (μM)	Inh %
**1**	– [c]	7.8 ± 5.0	–	5.1 ± 6.1
**2**	–	5.0 ± 3.4	–	11.3 ± 1.9 **
**3**	–	2.4 ± 3.1	–	7.5 ± 3.1
**4**	–	11.2 ± 3.8 *	–	3.6 ± 1.2 *
**5**	–	5.6 ± 6.2	–	10.0 ± 2.5 *
**6**	–	3.1 ± 3.1	–	5.6 ± 0.8 **
**7**	–	4.6 ± 3.6	–	3.9 ± 1.7
**9**	–	2.3 ± 2.8	–	4.3 ± 0.7 **
**11**	–	4.0 ± 4.4	–	5.4 ± 3.7
**12**	–	14.9 ± 6.2	–	8.7 ± 3.7
**13**	–	25.1 ± 7.5 *	–	0.3 ± 1.2
**15**	–	2.7 ± 1.0	–	5.5 ± 1.6 *
**18**	–	6.7 ± 3.3	–	19.5 ± 5.3 *
**26**	6.1 ± 0.3	69.9 ± 4.4 ***	–	11.8 ± 2.1 **
**29**	–	3.9 ± 3.1	–	9.1 ± 5.1
**32**	–	5.0 ± 1.0 **	–	2.8 ± 2.0
**33**	–	1.5 ± 1.2	–	3.9 ± 1.2 *
**34**	–	1.5 ± 0.7	–	4.7 ± 1.0 **
**46**	–	30.8 ± 6.6 **	–	13.3 ± 3.5 *
**47**	–	6.2 ± 0.5 ***	–	7.2 ± 5.2
**49**	–	10.1 ± 4.8	–	3.4 ± 2.7
**52**	–	4.2 ± 1.9	–	4.3 ± 2.3
**53**	–	5.9 ± 2.9	–	3.3 ± 2.4
**54**	4.5 ± 0.3	93.6 ± 3.3 ***	–	23.7 ± 1.1 ***
**55**	4.1 ± 0.2	99.0 ± 1.9 ***	3.8 ± 0.1	89.4 ± 4.5 ***
**56**	–	19.1 ± 7.3	–	27.4 ± 2.2 ***
**57**	5.0 ± 0.4	88.4 ± 5.3 ***	4.7 ± 0.4	89.9 ± 2.2 ***
**58**	–	9.7 ± 4.1	–	13.3 ± 3.7 *
**59**	–	3.4 ± 3.7	–	7.4 ± 4.0
**60**	–	7.9 ± 2.4 *	–	5.4 ± 3.4
**61**	–	6.5 ± 3.8	–	9.0 ± 3.3 *
**62**	–	8.6 ± 5.7	–	10.9 ± 3.3 *
**63**	9.3 ± 0.3	52.5 ± 1.2 ***	4.9 ± 0.2	75.2 ± 3.2 ***
**64**	–	14.2 ± 3.2 *	–	33.2 ± 3.7 ***
**65**	1.9 ± 0.2	89.3 ± 2.9 ***	6.4 ± 0.7	61.3 ± 4.7 ***
**66**	–	27.4 ± 7.5 *	7.7 ± 0.5	60.4 ± 2.3 ***
**67**	3.2 ± 0.1	100.0 ± 1.3 ***	4.1 ± 0.7	99.6 ± 7.6 ***
**68**	–	15.5 ± 5.6 *	–	4.3 ± 1.3 *
**69**	–	11.6 ± 2.9 *	–	14.6 ± 2.6 **
**70**	5.6 ± 0.9	85.2 ± 9.7 ***	–	46.5 ± 2.2 ***
**72**	–	10.4 ± 5.5	–	15.8 ± 4.0 *
**LY294002** [d]	1.0 ± 0.2		3.1 ± 0.7	

Results are presented as mean ± SEM (n=3). $*p < 0.05$, $**p < 0\ .01$, $***p < 0.001$ compared with the control (DMSO). [a] Concentration necessary for 50 % inhibition (IC_{50}). [b] Percentage of inhibition (Inh %) at 10 μM concentration. [c] Not determined. [d] A phosphatidylinositol-3-kinase inhibitor was used as a positive control.

Fig. S1. ^{1}H NMR spectrum of **1** (CD_3OD, 500 MHz).

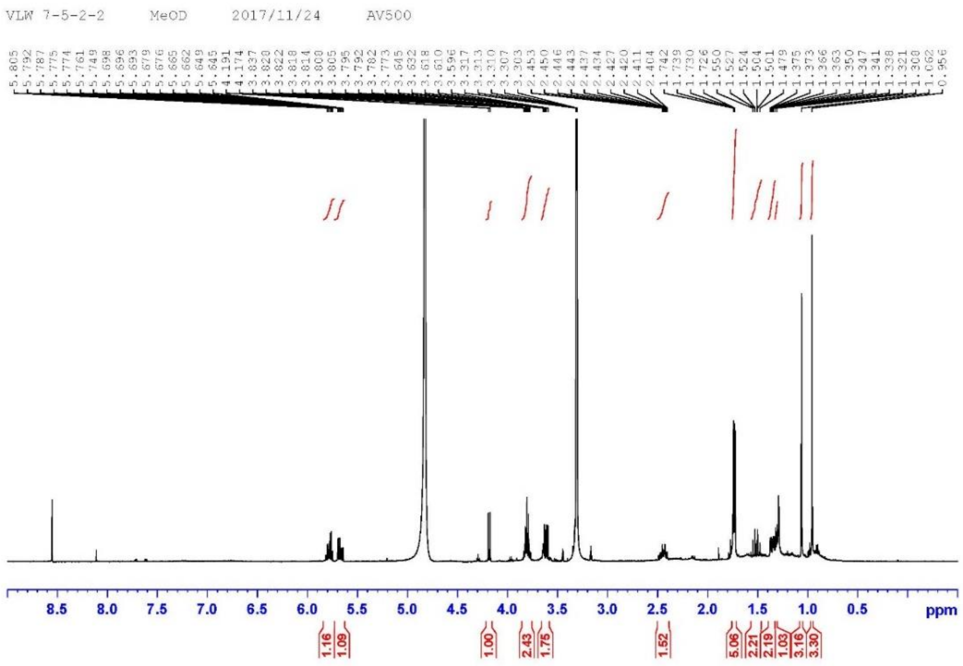

Fig. S2. ^{13}C and DEPT NMR spectrum of **1** (CD_3OD, 125 MHz).

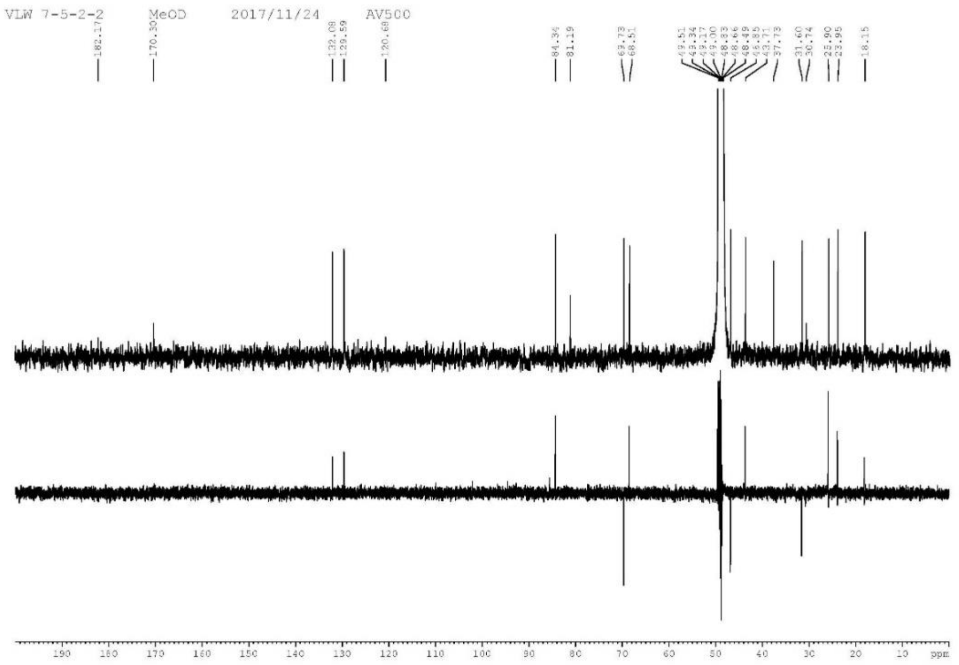

Fig. S3. COSY spectrum of **1** (CD_3OD, 500 MHz).

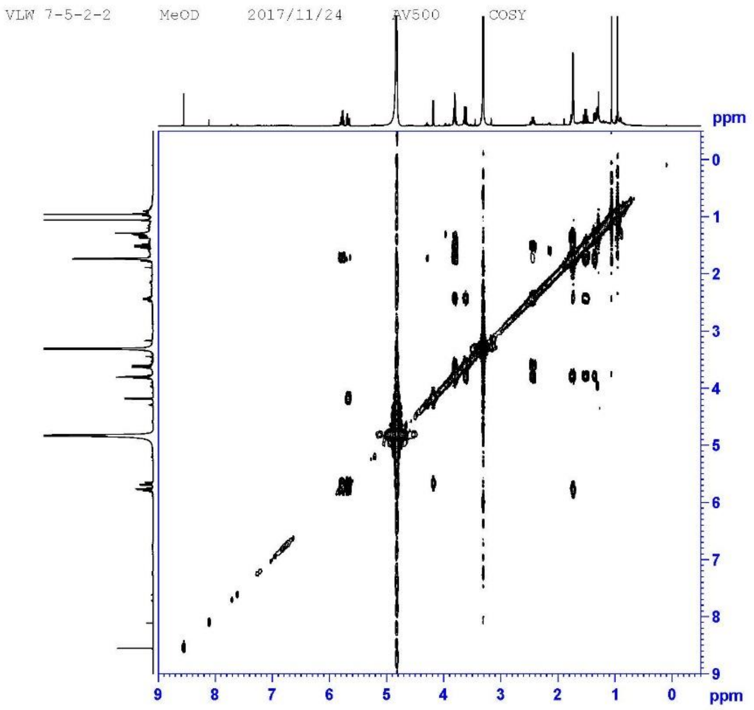

Fig. S4. HMQC spectrum of **1** (CD_3OD, 500 MHz).

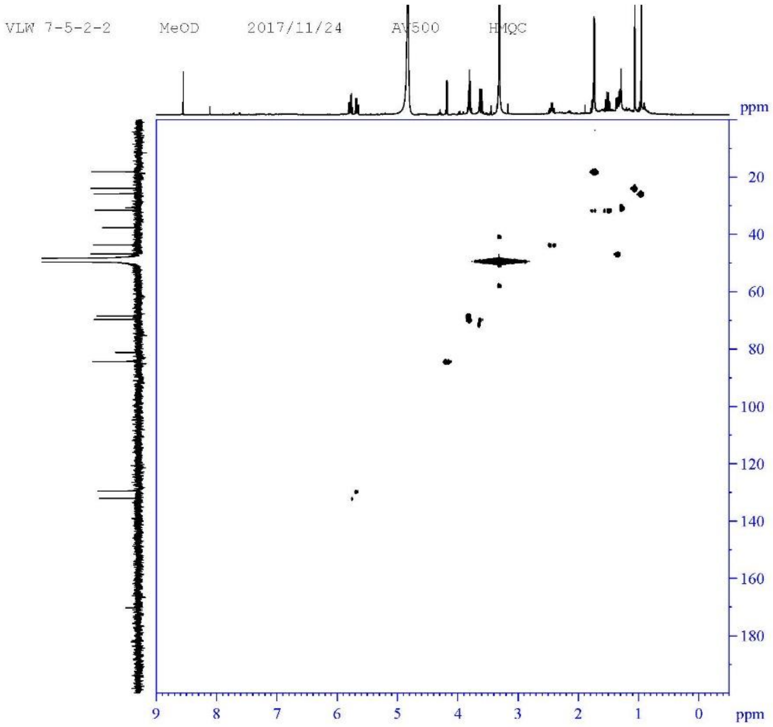

Fig. S5. HMBC spectrum of **1** (CD_3OD, 500 MHz).

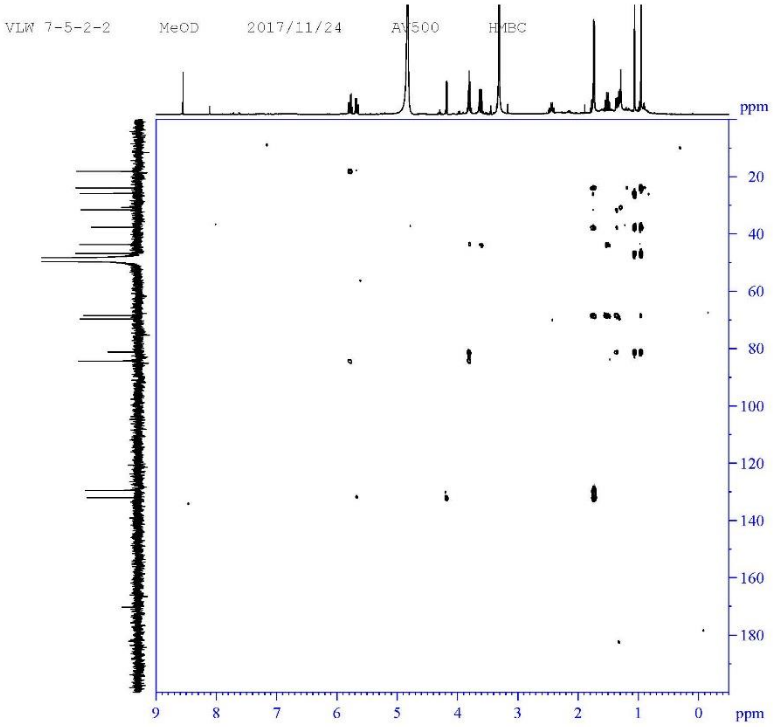

Fig. S6. NOESY spectrum of **1** (CD_3OD, 500 MHz).

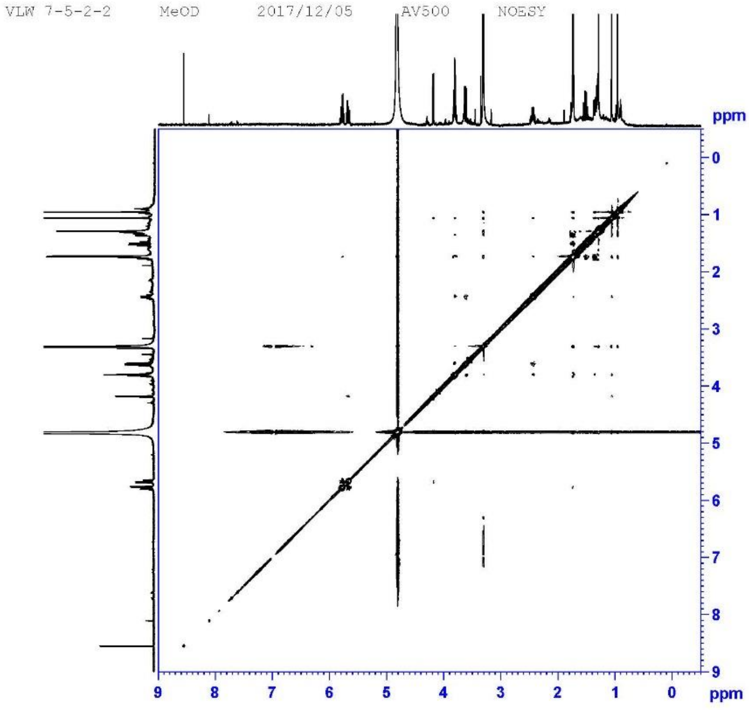

Fig. S7. HRMS spectrum of **1**.

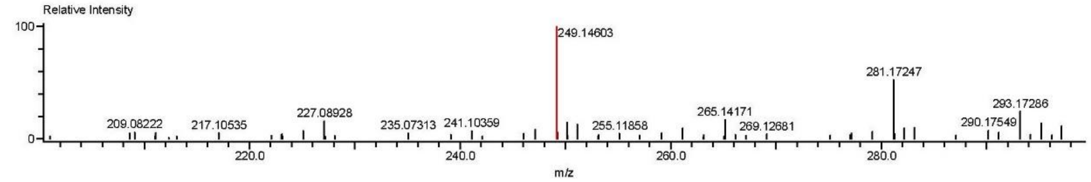

Mass	Intensity	Calc. Mass	Mass Difference [mDa]	Mass Difference [ppm]	Possible Formula	Unsaturation Number
249.14603	18321.00	249.14666	-0.63	-2.53	$^{12}C_{13}{}^{1}H_{22}{}^{23}Na_{1}{}^{16}O_{3}$	2.5

Fig. S8. ^{1}H NMR spectrum of **2** (CD_3OD, 500 MHz).

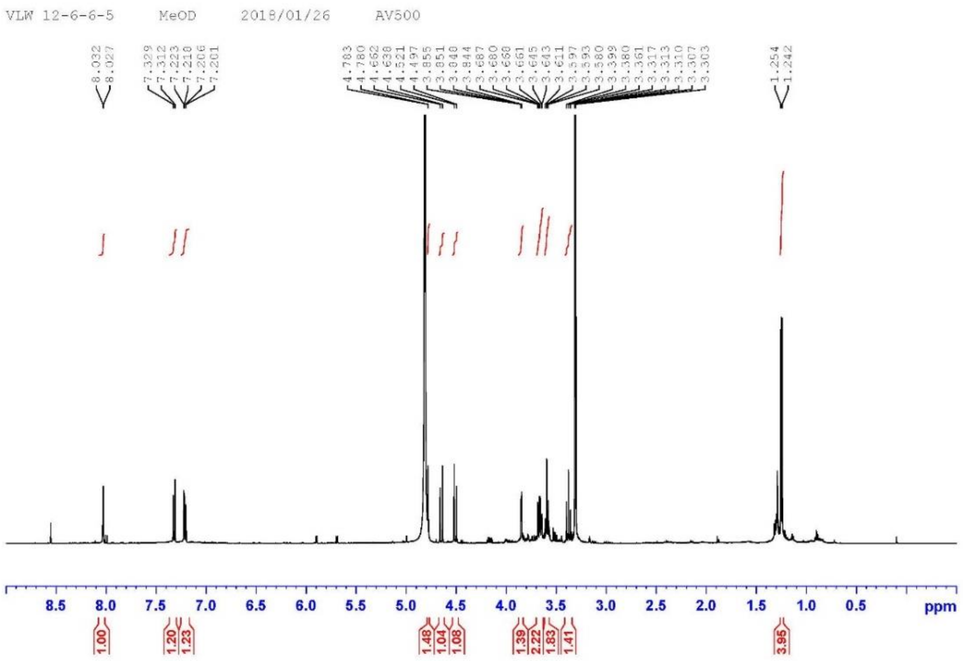

Fig. S9. ^{13}C and DEPT NMR spectrum of **2** (CD_3OD, 125 MHz).

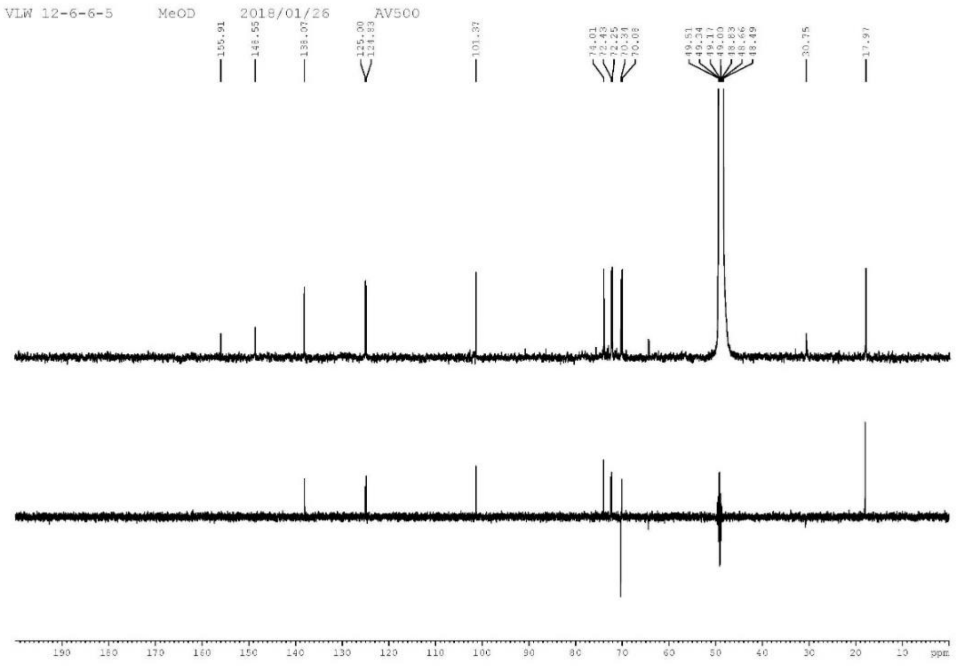

Fig. S10. COSY spectrum of **2** (CD_3OD, 500 MHz).

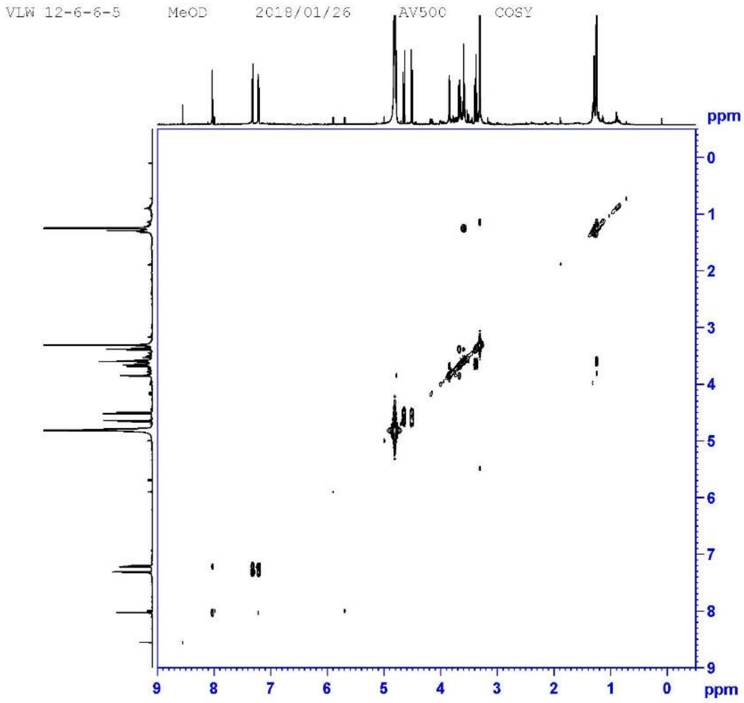

Fig. S11. HMQC spectrum of **2** (CD_3OD, 500 MHz).

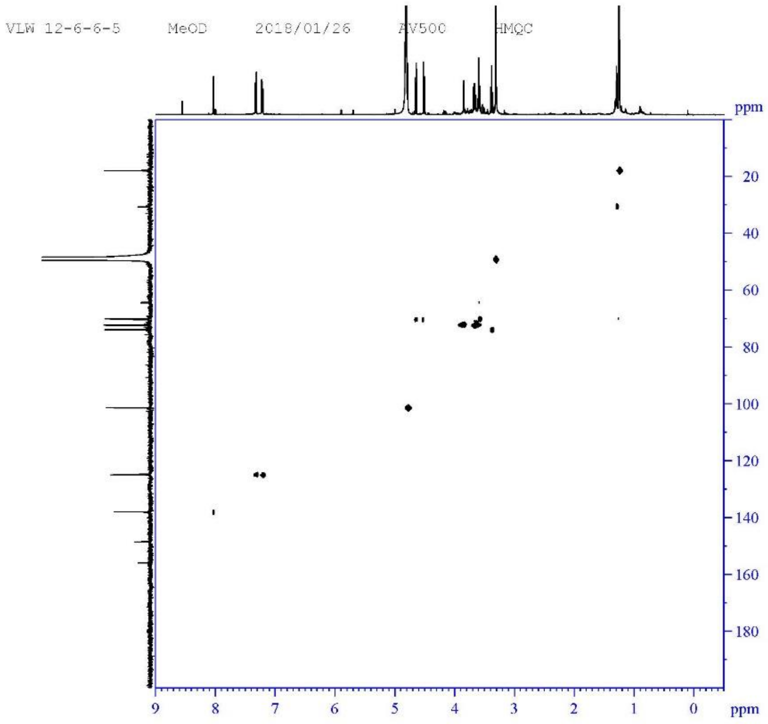

Fig. S12. HMBC spectrum of **2** (CD_3OD, 500 MHz).

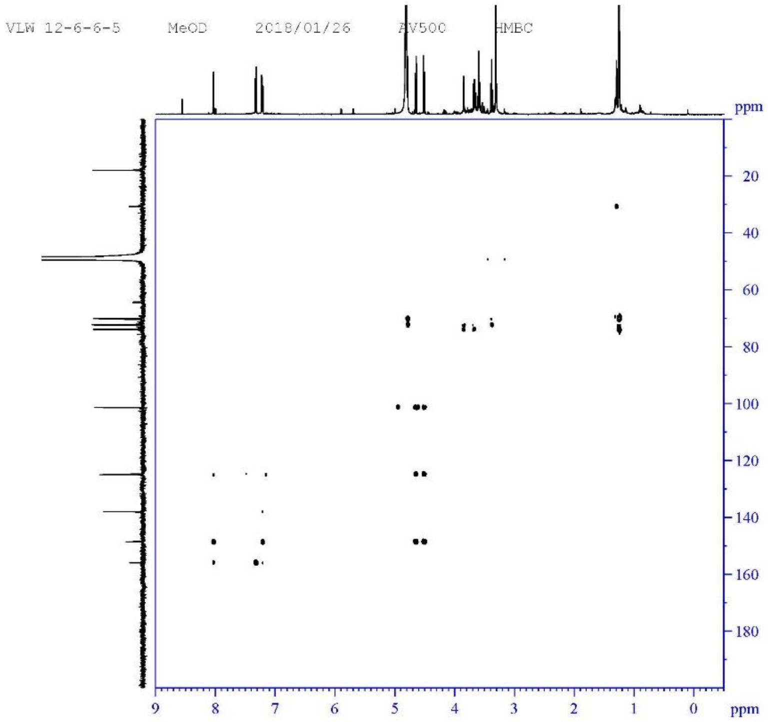

Fig. S13. NOESY spectrum of **2** (CD_3OD, 500 MHz).

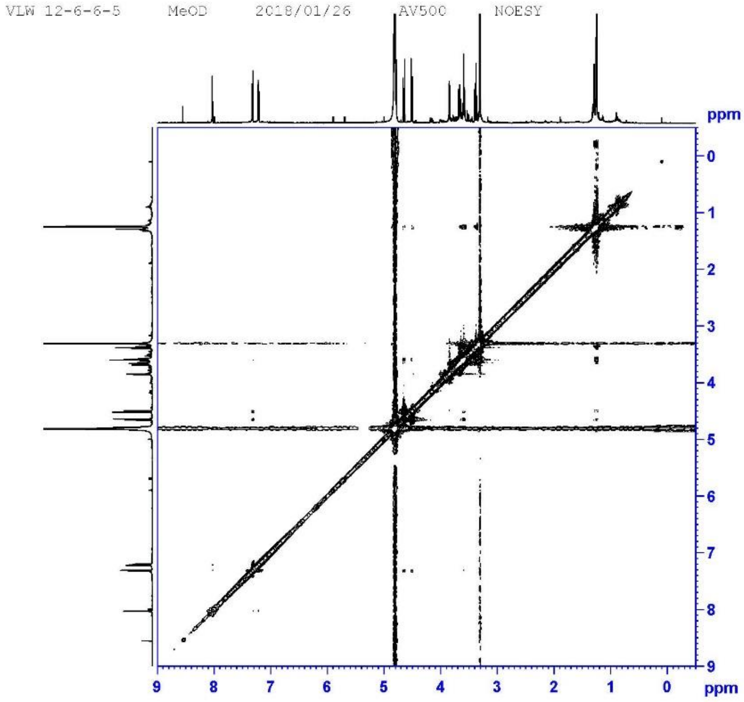

Fig. S14. MS/HRMS spectrum of **2**.

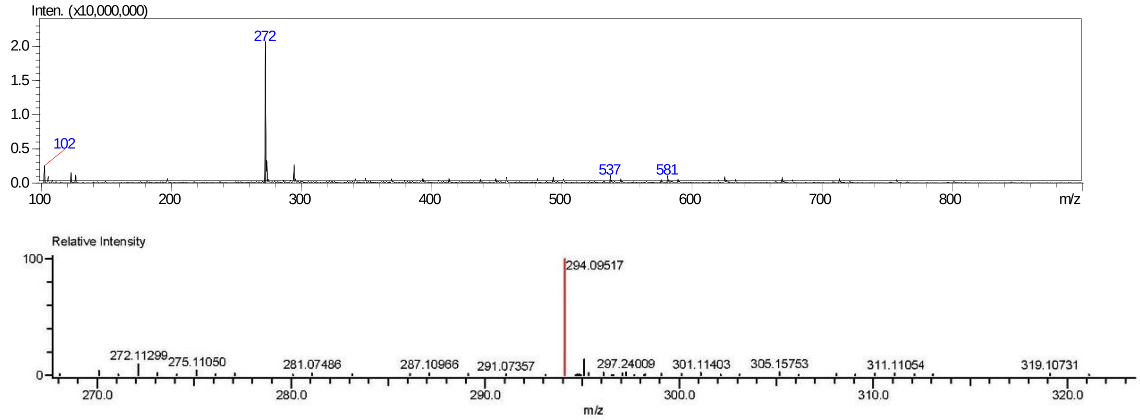

Mass	Intensity	Calc. Mass	Mass Difference [mDa]	Mass Difference [ppm]	Possible Formula	Unsaturation Number
294.09517	112665.40	294.09536	-0.18	-0.62	$^{12}C_{12}{}^{1}H_{17}{}^{14}N_{1}{}^{23}Na_{1}{}^{16}O_{6}$	4.5

Fig. S15. ^{1}H NMR spectrum of **3** (CD_3OD, 400 MHz).

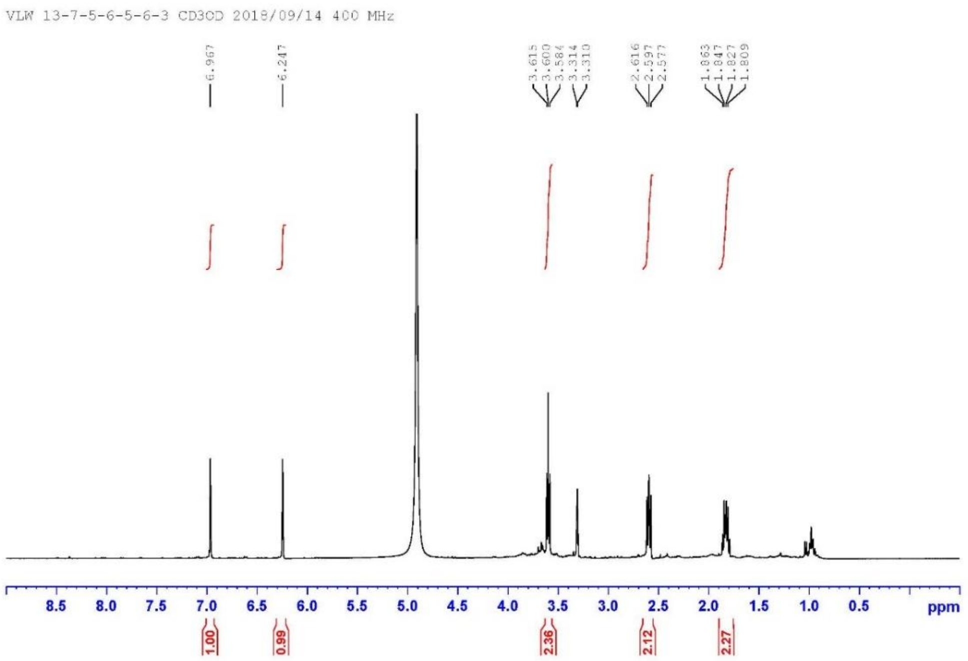

Fig. S16. ^{13}C and DEPT NMR spectrum of **3** (CD_3OD, 100 MHz).

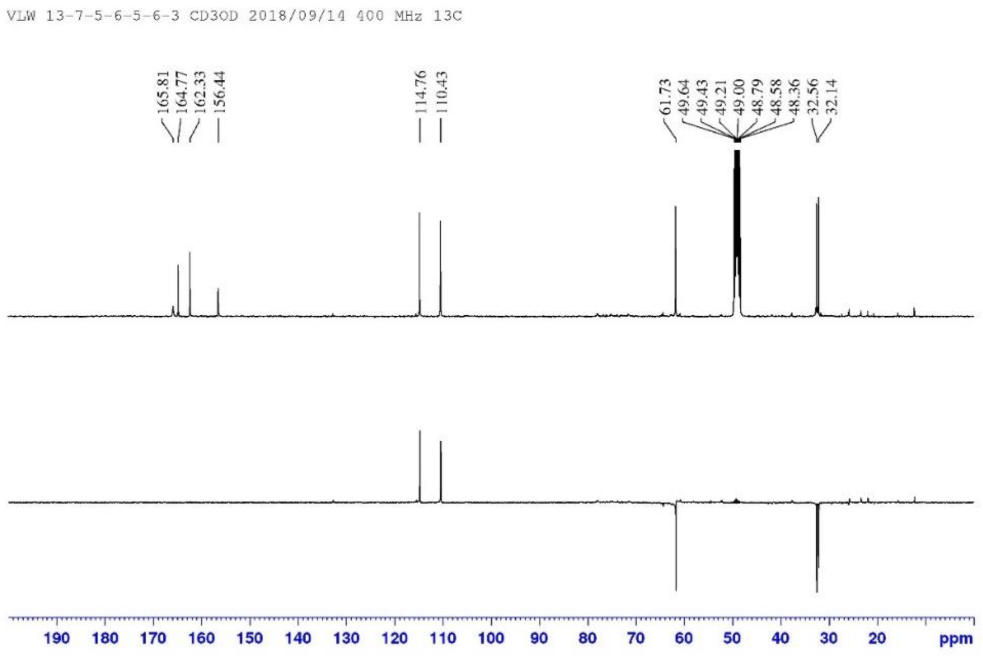

Fig. S17. COSY spectrum of **3** (CD_3OD, 400 MHz).

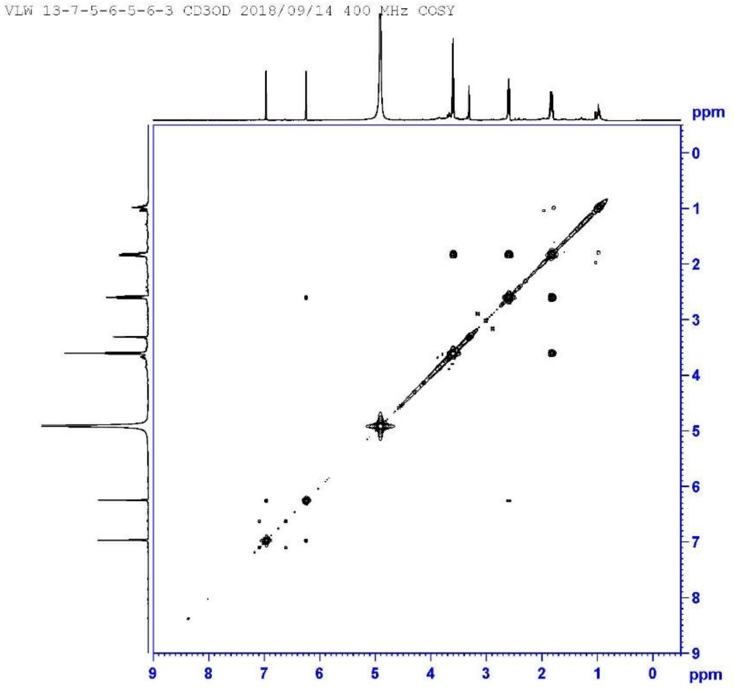

Fig. S18. HMQC spectrum of **3** (CD_3OD, 400 MHz).

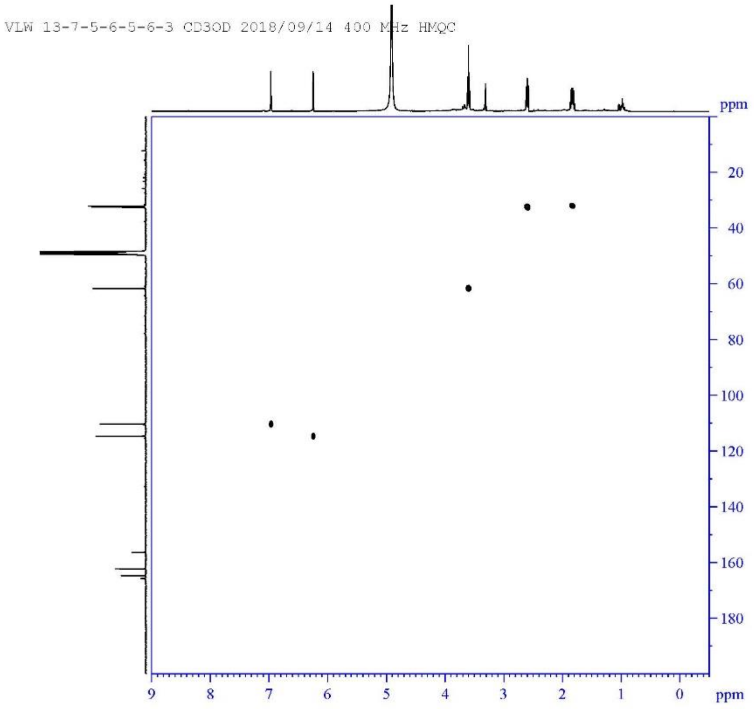

Fig. S19. HMBC spectrum of **3** (CD_3OD, 400 MHz).

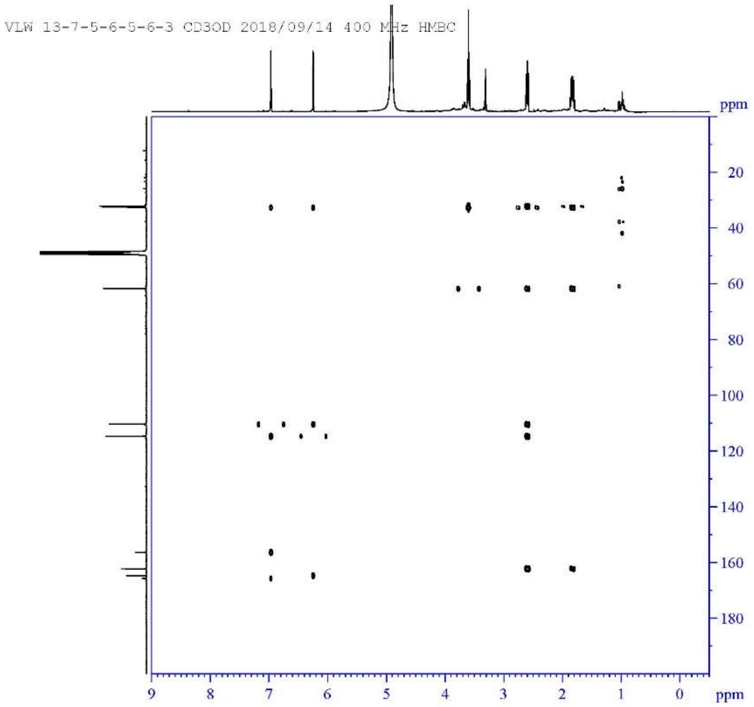

Fig. S20. NOESY spectrum of **3** (CD_3OD, 400 MHz).

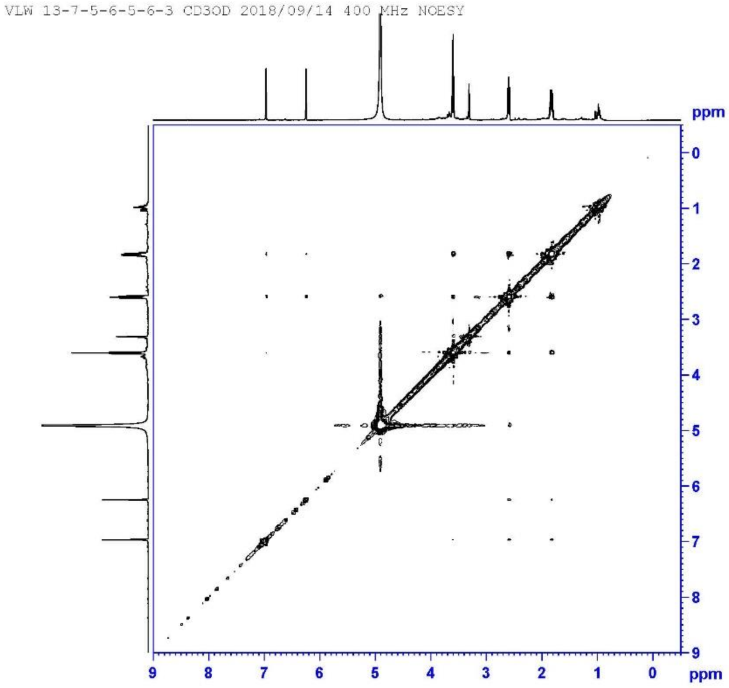

Fig. S21. MS/HRMS spectrum of **3**.

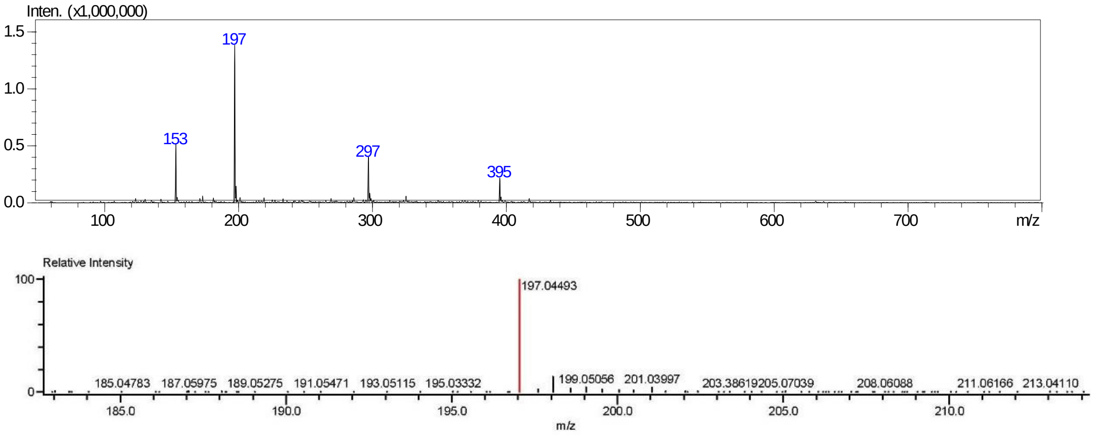

Mass	Intensity	Calc. Mass	Mass Difference [mDa]	Mass Difference [ppm]	Possible Formula	Unsaturation Number
197.04493	4128391.25	197.04500	-0.07	-0.36	$^{12}C_9{}^{1}H_9{}^{16}O_5$	5.5

Fig. S22. ^{1}H NMR spectrum of **4** (CD_3OD, 400 MHz).

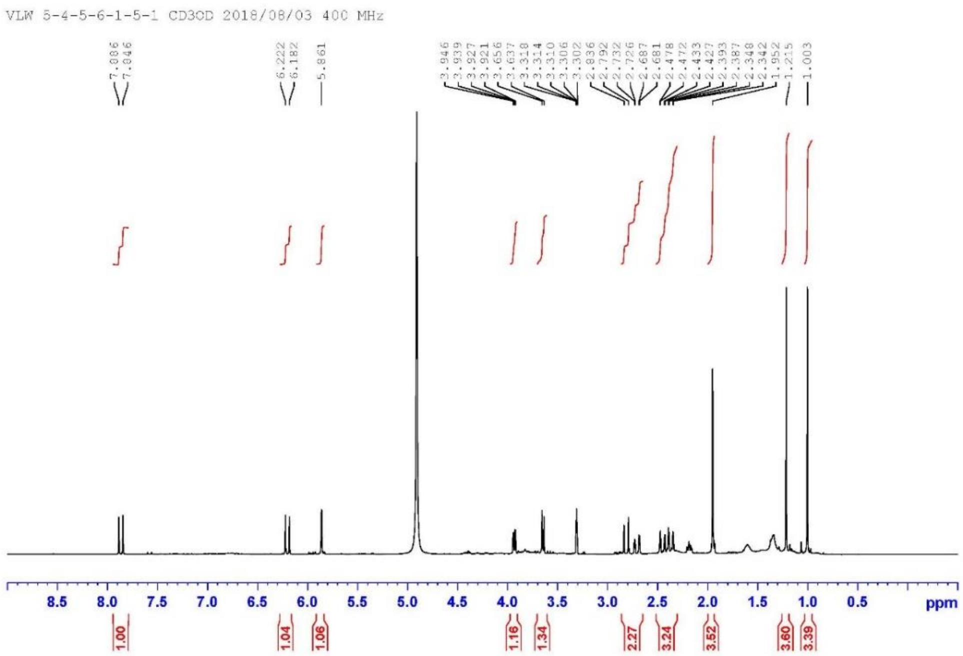

Fig. S23. ^{13}C and DEPT NMR spectrum of **4** (CD_3OD, 100 MHz).

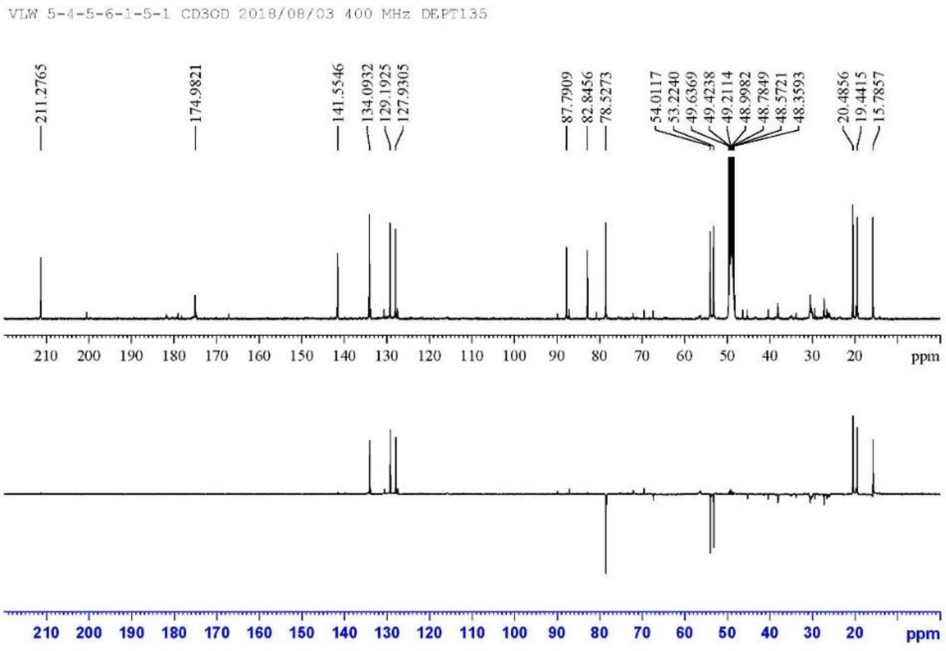

Fig. S24. COSY spectrum of **4** (CD_3OD, 400 MHz).

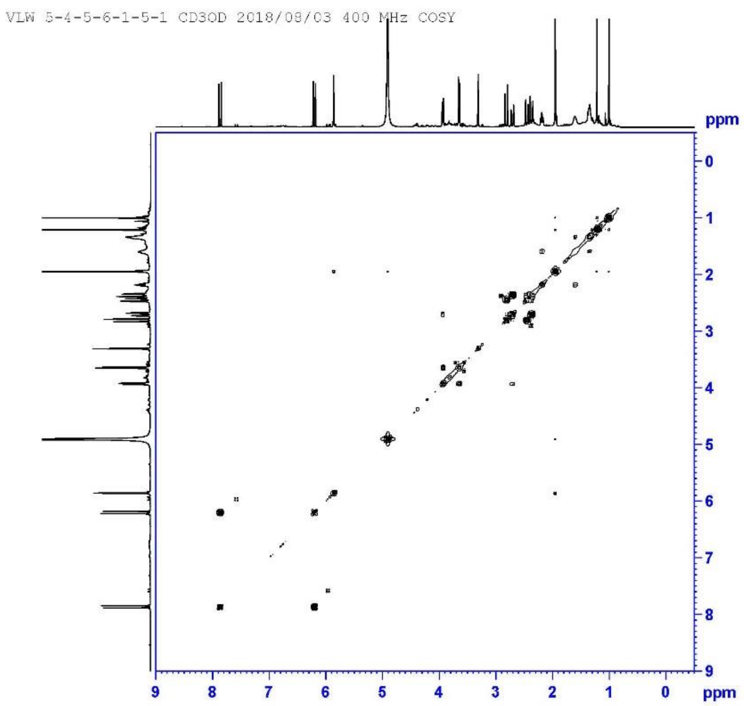

Fig. S25. HMQC spectrum of **4** (CD_3OD, 400 MHz).

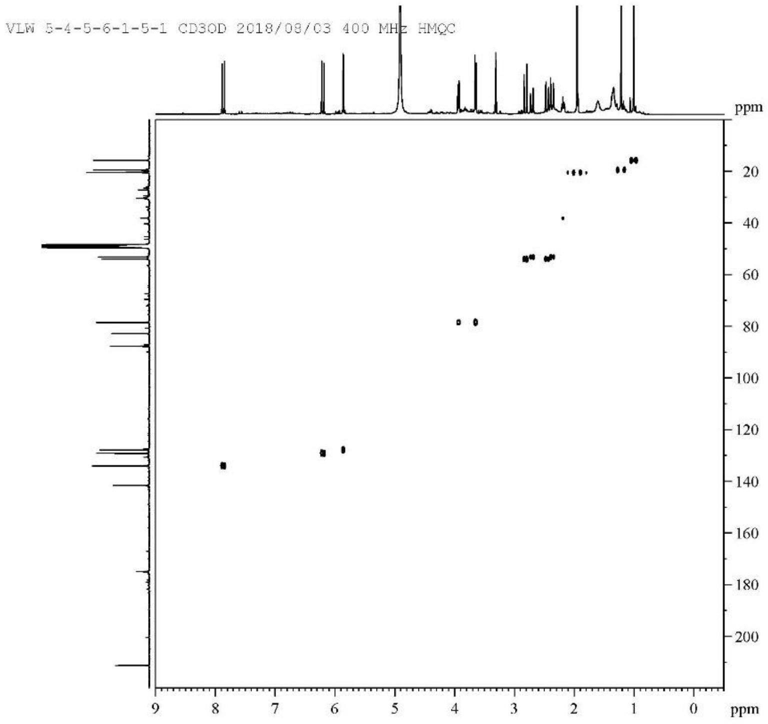

Fig. S26. HMBC spectrum of **4** (CD_3OD, 400 MHz).

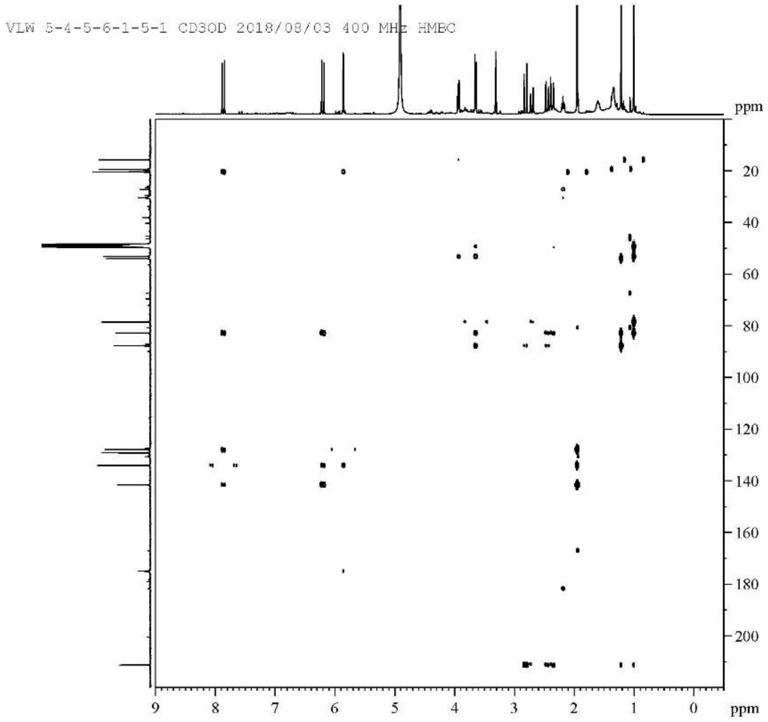

Fig. S27. NOESY spectrum of **4** (CD_3OD, 400 MHz).

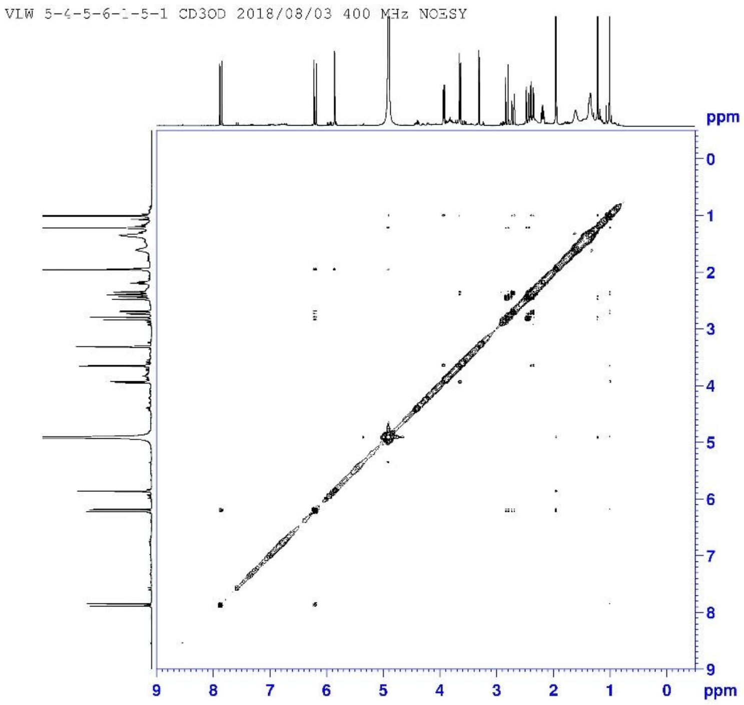

Fig. S28. ^{1}H NMR spectrum of **4** after acidification (CD_3OD, 500 MHz).

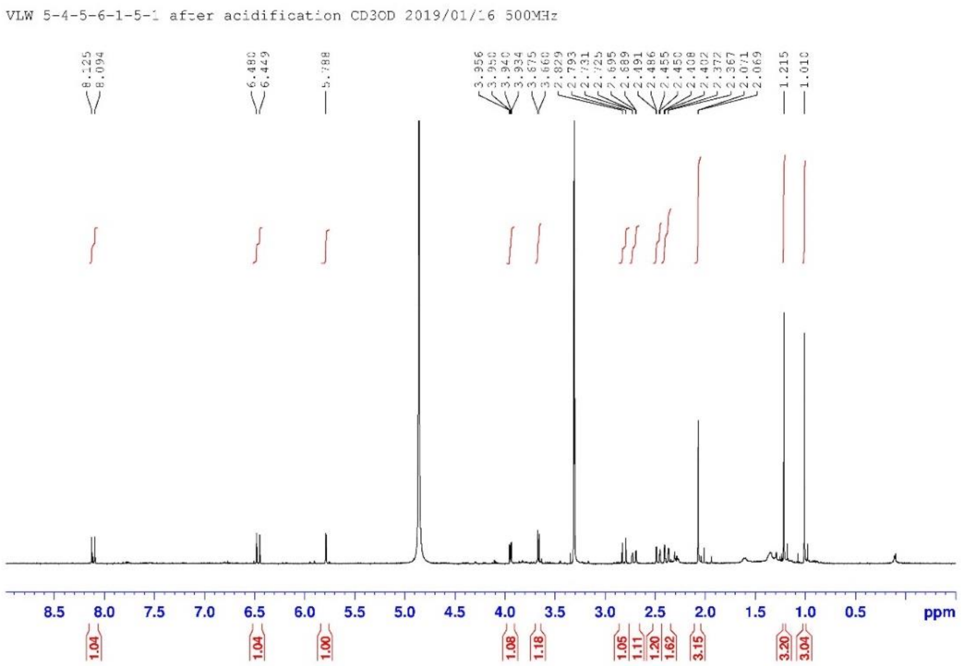

Fig. S29. MS/HRMS spectrum of **4**.

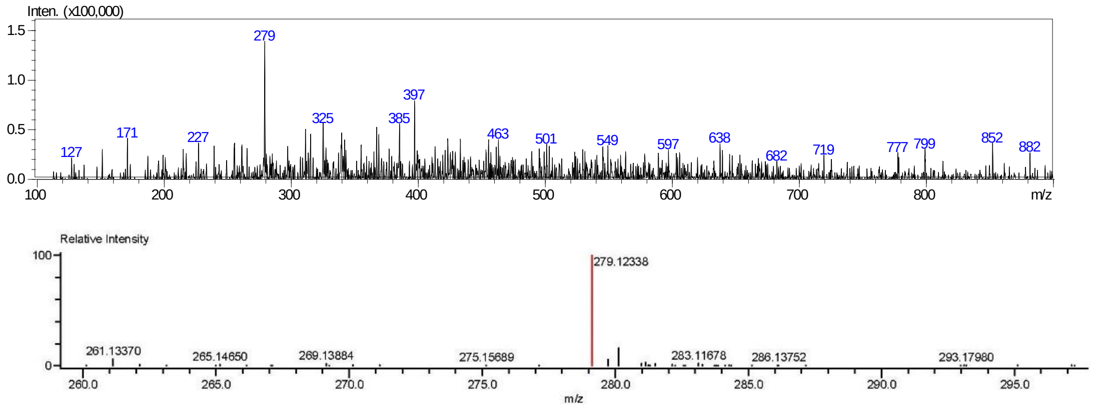

Mass	Intensity	Calc. Mass	Mass Difference [mDa]	Mass Difference [ppm]	Possible Formula	Unsaturation Number
279.12338	279560.75	279.12325	0.13	0.46	$^{12}C_{15}{}^{1}H_{19}{}^{16}O_5$	6.5

Fig. S30. ^{1}H NMR spectrum of **5** (CD_3OD, 400 MHz).

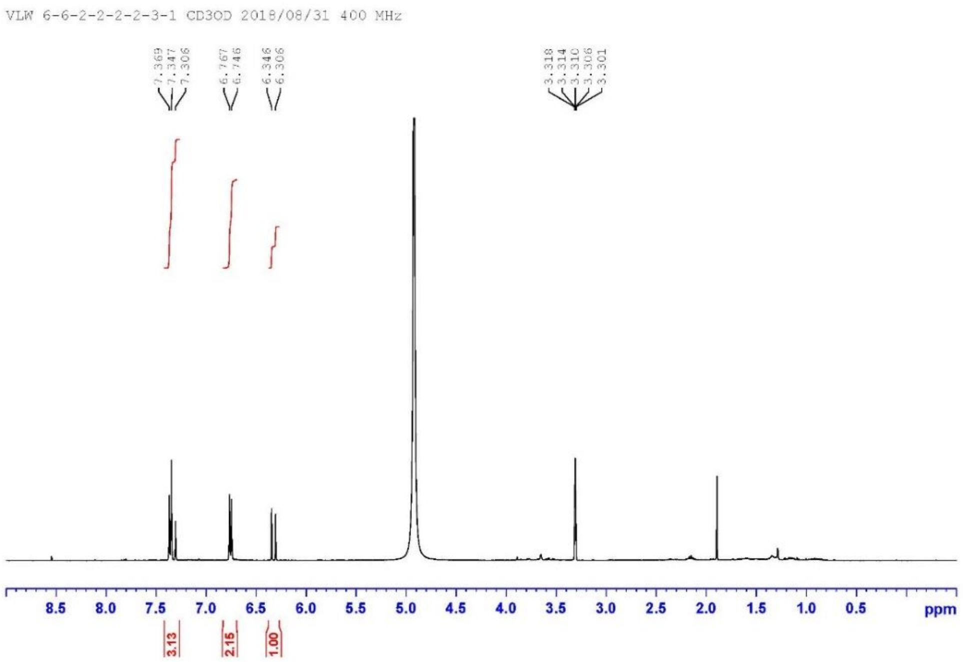

Fig. S31. ^{13}C and DEPT NMR spectrum of **5** (CD_3OD, 100 MHz).

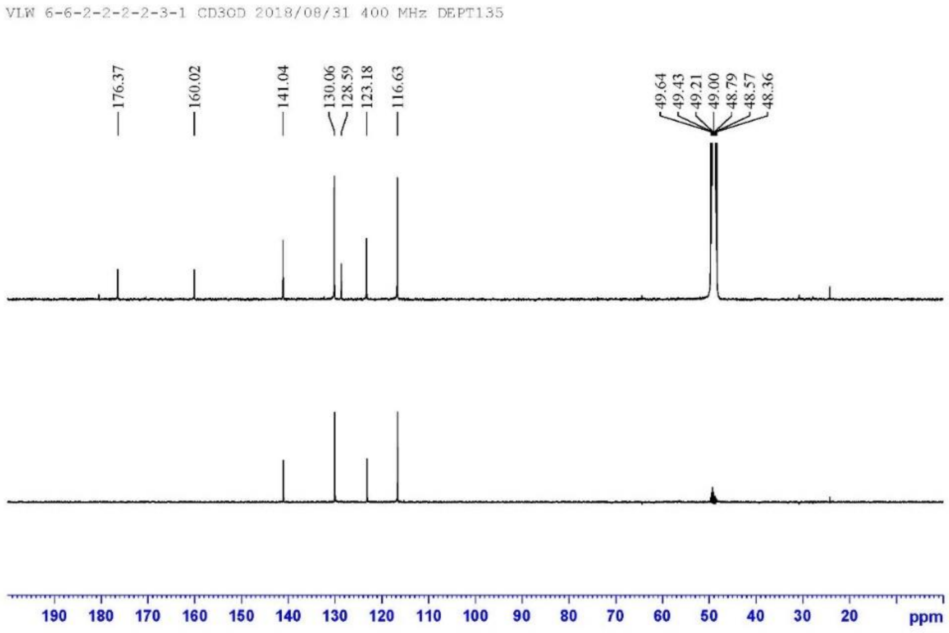

Fig. S32. COSY spectrum of **5** (CD_3OD, 400 MHz).

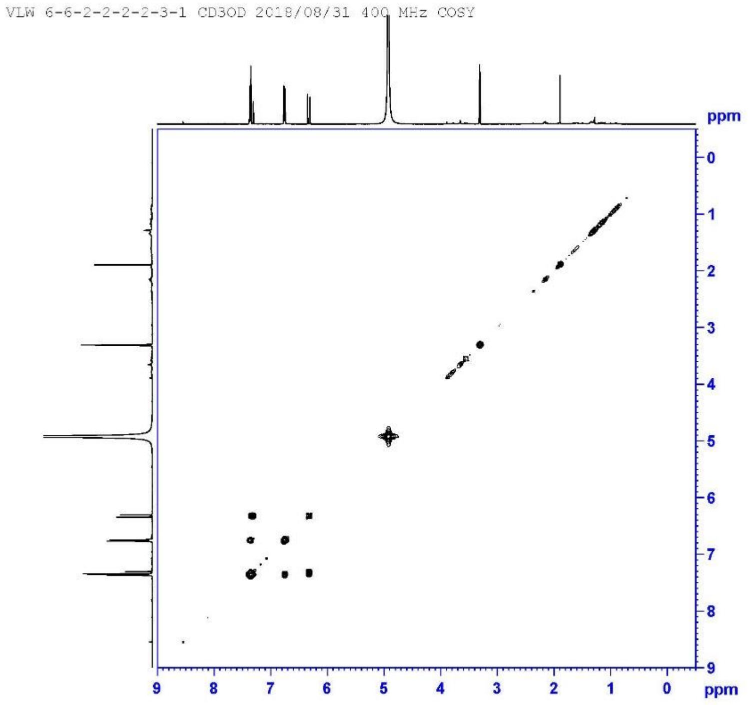

Fig. S33. HMQC spectrum of **5** (CD_3OD, 400 MHz).

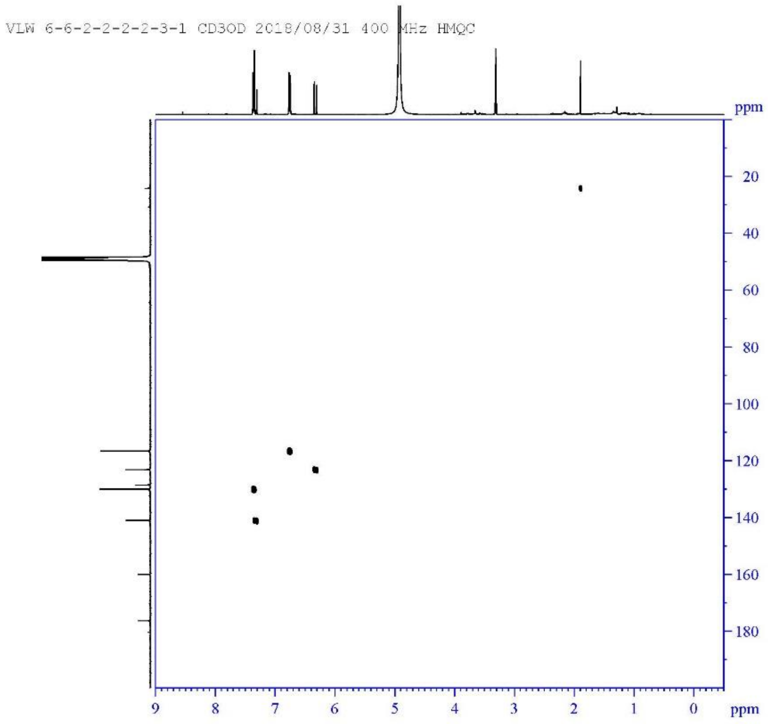

Fig. S34. HMBC spectrum of **5** (CD_3OD, 400 MHz).

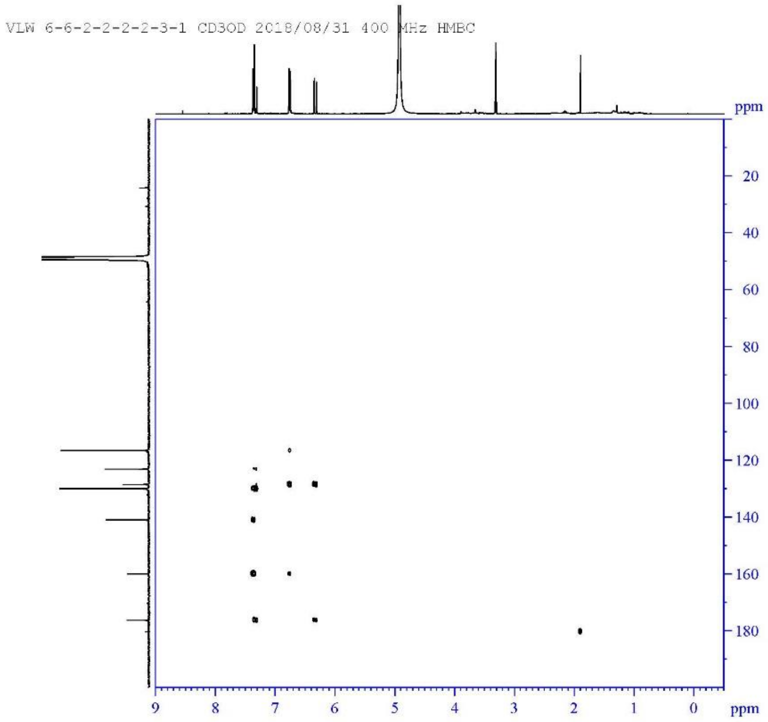

Fig. S35. NOESY spectrum of **5** (CD_3OD, 400 MHz).

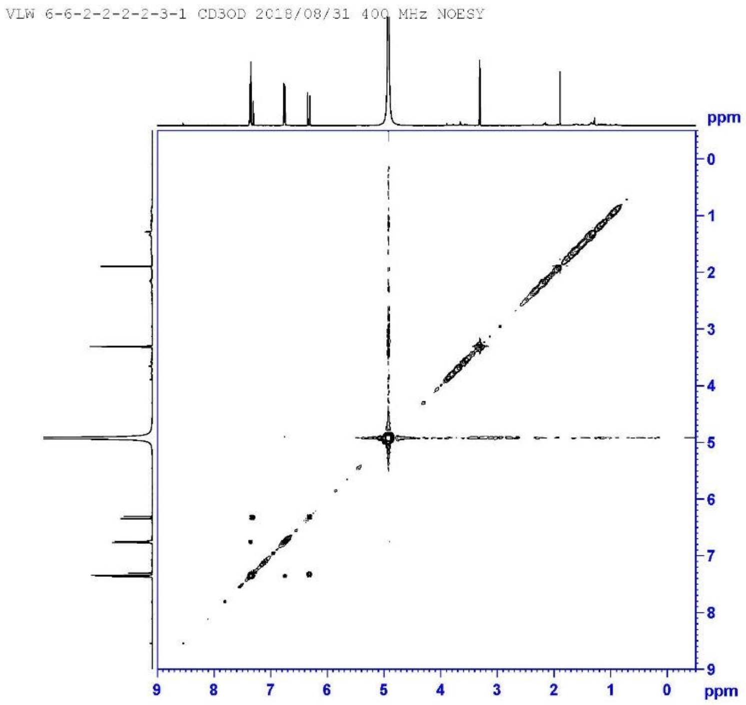

Fig. S36. ^{1}H NMR spectrum of **5** after acidification (CD_3OD, 500 MHz).

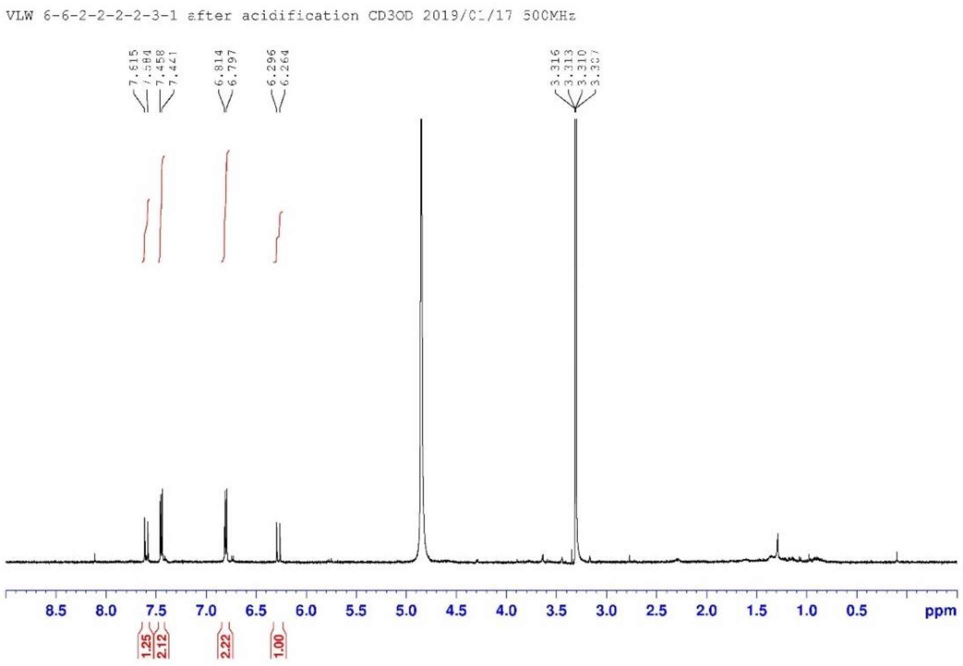

Fig. S37. MS/HRMS spectrum of **5**.

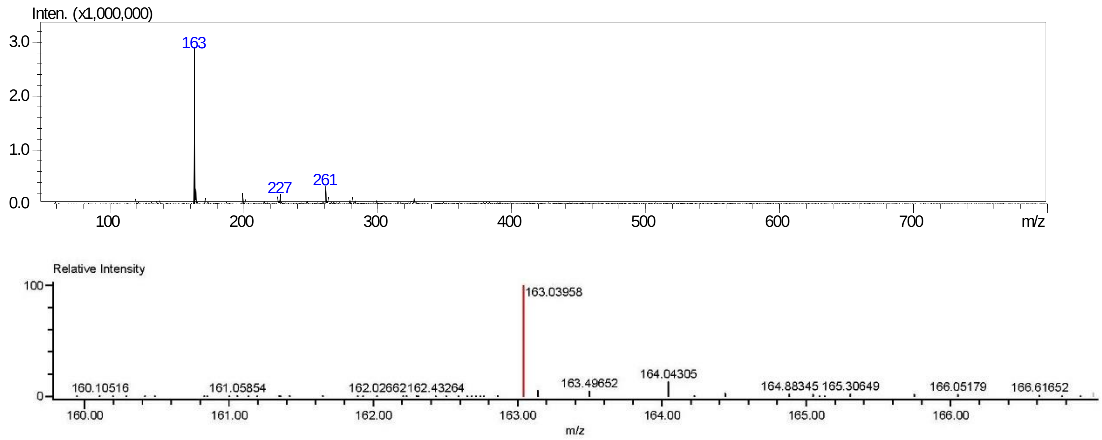

Mass	Intensity	Calc. Mass	Mass Difference [mDa]	Mass Difference [ppm]	Possible Formula	Unsaturation Number
163.03958	1705178.50	163.03952	0.06	0.37	$^{12}C_9{}^{1}H_7{}^{16}O_3$	6.5
